# Th40 cells (CD4^+^CD40^+^ Tcells) drive a more severe form of Experimental Autoimmune Encephalomyelitis than conventional CD4 T cells

**DOI:** 10.1371/journal.pone.0172037

**Published:** 2017-02-13

**Authors:** Gisela M. Vaitaitis, Martin G. Yussman, Dan M. Waid, David H. Wagner

**Affiliations:** 1 Department of Medicine, University of Colorado Denver, Anschutz Medical Campus, Aurora, Colorado, United States of America; 2 Webb-Waring Center, University of Colorado Denver, Anschutz Medical Campus, Aurora, Colorado, United States of America; University of British Columbia, CANADA

## Abstract

CD40-CD154 interaction is critically involved in autoimmune diseases, and CD4 T cells play a dominant role in the Experimental Autoimmune Encephalomyelitis (EAE) model of Multiple Sclerosis (MS). CD4 T cells expressing CD40 (Th40) are pathogenic in type I diabetes but have not been evaluated in EAE. We demonstrate here that Th40 cells drive a rapid, more severe EAE disease course than conventional CD4 T cells. Adoptively transferred Th40 cells are present in lesions in the CNS and are associated with wide spread demyelination. Primary Th40 cells from EAE-induced donors adoptively transfer EAE without further *in-vitro* expansion and without requiring the administration of the EAE induction regimen to the recipient animals. This has not been accomplished with primary, non-TCR-transgenic donor cells previously. If co-injection of Th40 donor cells with Freund’s adjuvant (CFA) in the recipient animals is done, the disease course is more severe. The CFA component of the EAE induction regimen causes generalized inflammation, promoting expansion of Th40 cells and infiltration of the CNS, while MOG-antigen shapes the antigen-specific TCR repertoire. Those events are both necessary to precipitate disease. In MS, viral infections or trauma may induce generalized inflammation in susceptible individuals with subsequent disease onset. It will be important to further understand the events leading up to disease onset and to elucidate the contributions of the Th40 T cell subset. Also, evaluating Th40 levels as predictors of disease onset would be highly useful because if either the generalized inflammation event or the TCR-honing can be interrupted, disease onset may be prevented.

## Introduction

Multiple sclerosis (MS) is an inflammatory demyelinating disease of the central nervous system (CNS) that has an undetermined etiology. An autoimmune component is critical to disease development; demonstrated largely by the Experimental Autoimmune Encephalomyelitis (EAE) model, the accepted model for MS. In MS and EAE, sclerotic lesions form in the brain and spinal cord that involves infiltration by culprit inflammatory cells, including macrophages [1], mast cells [2], and T cells [3–5]. EAE is induced in mice by injecting CNS associated antigens [6], but major histocompatibility complex (MHC) haplotype, antigen specificity, and concentration dictate whether chronic or relapsing-remitting disease occurs [7]. Transfer of enriched antigen-specific CD4^+^ T cells from EAE induced donors facilitates disease induction in recipients [8]. However, for donor cells to transfer disease, it is necessary to first further expand the cells *in-vitro* in the presence of more antigen and IL-12 before transfer of the cells or, alternatively, to administer the full EAE induction regimen to the recipient mice [8–11]. This is different from, for example, the T1D model where primary donor T cells transfer disease without further manipulation [12, 13]. CD8^+^ T cells also play a role in EAE [7] but cause different symptoms than those induced by CD4^+^ T cells [14]. CD4^+^ T cell driven EAE depends upon a Th1 phenotype, in combination with Th17 cells, and thus far is thought to rely on co-stimulus through CD28, a molecule recognized as necessary for T cell activation [15]. Interestingly however, while CD28 knockout mice challenged once with EAE immunization do not develop disease, mice that are re-challenged experience a rapid and severe form of EAE, independently of CD28 co-stimulus. The onset is prevented completely by blocking CD40-CD154 signaling [15]. Preventing CD40-CD154 signaling in wild type mice, either by blocking CD154 [15] or by blocking CD40 [2], decreases the severity and delays the onset of EAE. This suggests a determinant role for CD40, perhaps involving T cells.

Through extensive work, we identified a subset of CD4 T cells that is characterized by CD40 expression. Because these cells express IFNγ and IL-17 concomitantly, we termed them Th40 cells [16]. Like regulatory T cells (Treg), Th40 cells develop in the thymus [17]. While Th40 cells are present in non-autoimmune strains (up to 25%), they expand to about 60% of the CD4 compartment in non-obese diabetic (NOD) mice, a model of type I diabetes (T1D) and, coincidentally, a model for relapsing-remitting EAE [18]. Primary, peripheral Th40 cells successfully transfer T1D without additional requirements [12, 13, 19]. Interestingly, Tregs are less able to regulate Th40 cells than other, CD40-negative effector T cells [17]. Translational studies revealed that non-autoimmune, human subjects maintain peripheral levels of Th40 cells at up to 30% of the CD4 T cell compartment [20, 21]. Similar to the T1D animal model, human subjects with MS [20] or T1D [21] have an expansion of Th40 cells (up to 50% or more of the CD4 compartment) in peripheral blood compared to controls. Subjects with type 2 diabetes, a non-autoimmune, chronic disease, do not demonstrate that expansion [20, 21]. In a double blind study, Th40 cell expansion was more predictive of T1D than HLA-DR haplotype and those cells were highly responsive to T1D autoantigens [21]. The same was true of Th40 cells in MS [20]. In this reverse translational study, we address the role of Th40 cells in EAE and further define phases of the disease, which may in turn lead to better understanding of MS.

## Materials and methods

### Mice

C57BL/6 mice were from Taconic (Hudson, NY, USA). B6.CB17-Prkdc^scid^ mice were from Jackson Laboratories (Bar Harbor, ME, USA). Animals were housed at the University of Colorado Denver Anschutz Medical Campus AALAC approved Vivarium. This study was carried out in strict accordance with the recommendations in the Guide for the Care and Use of Laboratory Animals of the National Institutes of Health. The protocol was approved by the Institutional Animal Care and Use Committee at the University of Colorado Denver (Protocol number: B-55814(01)1E). All efforts were made to minimize suffering and euthanasia was performed using carbon-dioxide followed by either cervical dislocation or exsanguination.

### Antibodies and reagents

For flow cytometry: Anti-IFNγ (XMG1.2), anti-IL17A (eBio1787), anti-CD3 (145.2C11), anti-CD28 (37.51), anti-CD8 (53–6.7), anti-CD62L (MEL-14), anti-CD44 (IM7), anti-CD69 (H1.2F3), appropriate isotype control antibodies, and permeabilization buffer for intracellular stains were from eBioscience, Inc. (San Diego, CA, USA). TCR Vα and Vβ antibodies, and anti-CD4 (H129.19) were from BD Biosciences (San Jose, CA, USA). For cell purification/sorting: Lympholyte-M was from CedarLane (Burlington, NC, USA). Anti-CD8-, anti-CD4-, anti-MHCII-, anti-CD11b-, and streptavidin-microbeads were from Miltenyi Biotec (Auburn, CA, USA). Anti-CD40, 1C10 [22], was produced in-house. Anti-CD19-biotin, anti-CD11c-biotin, and anti-CD25-biotin were from eBioscience, Inc. (San Diego, CA, USA). Antibodies for western blot for CD40 (sc-975), CD4 (sc-1140) and CD3 (sc-1127) were from Santa Cruz Biotechnology (Dallas, TX, USA) and for TCRβ (H57-597) was produced in-house. Western blot detection reagent, ECL Prime was from GE Healthcare (Pittsburgh, PA, USA). For EAE induction: M. Tuberculosis H37 RA was from Becton Dickinson, and Company (Franklin Lakes, NJ, USA). Pertussis toxin (PT), Lipopolysaccharide (LPS), and polyinosinic:polycytidylic acid (poly I:C) were from Sigma-Aldrich^®^ (St. Louis, MO, USA). All other reagents were from Sigma-Aldrich^®^.

### Induction of EAE

Female 8–12 week old C57BL/6 mice were immunized subcutaneously on the upper back/neck with 100 μl completely emulsified MOG_35-55_ (100 μg) and complete Freund’s adjuvant (CFA; 200 μg M. Tuberculosis H37 RA in incomplete Freund’s adjuvant (mineral oil)), followed by PT (200 ng in 100 μl PBS) injections as described [9]. In some experiments MOG_35-55_ was omitted and in others both MOG_35-55_ and CFA was omitted and instead 200 μg LPS was emulsified in 100 μl mineral oil and injected subcutaneously. In yet other experiments 200 μg poly I:C in 100 μl PBS was injected intraperitoneally every 4 days for a total of 3 times.

All mice were monitored daily for disease and scored: 0, no abnormality; 1, limp tail or hind limb weakness; 2, limp tail and hind limb weakness; 3, partial hind limb paralysis; 4, complete hind limb paralysis; 5, moribund state. The data are reported as the mean daily clinical score for all animals in each group. Mice were euthanized if reaching a level 5 or if losing more than 20% of their bodyweight.

### Disease transfer experiments

Spleens from EAE induced mice were harvested 8 days post immunization, homogenized in Red Blood Cell Lysing Buffer and the cells were pelleted. Th40 cells were sorted as described [23] then 6 x 10^7^ cells were transferred into C57BL/6.scid mice by intraperitoneal injection followed by a subcutaneous injection of CFA. In other transfer experiments Th40 cells were sorted as above then further depleted on CD11b, CD11c, and CD19 (to further ensure removal of B-cells, macrophages and dendritic cells) then transferred into C57BL/6.scid mice as above. In addition, CD4^+^(CD40^(surface)-^) T cells were purified by removing CD8, MHCII, CD40 (1C10, 4F11, HM40-3, and FGK-45), CD11b, CD11c, CD19, and CD25 then 6 x 10^7^ cells were transferred into C57BL/6.scid mice as above.

### Mononuclear cell isolation

Mononuclear cells were isolated from the brain, spinal cord, spleen, and peripheral draining lymph nodes (dLN; cervical and deep cervical) of mice perfused with PBS. Brain and spinal cord were homogenized in HBSS, and mononuclear cells were isolated using 37%/70% discontinuous Percoll gradients. For CD40 and CD3 western blots, as well as for stains on cells recovered from brain and spinal cord, the recovered cells were MHCII and CD11b depleted to remove B cells, macrophages, and any contaminating microglial cells. The spleen and dLN (superficial and deep cervical) were homogenized in Red Blood Cell Lysing Buffer or PBS containing 2mM EDTA and 5% BSA, respectively. Lymphocytes were then isolated on Lympholyte-M (CEDARLANE, Ontario, Canada).

### Cell staining and flow cytometry

Purified cells were stained and flow cytometry was conducted using a MACSQuant Analyzer (Miltenyi Biotec, Auburn, CA, USA). Gates were set from isotype controls such that in the isotype stained sample, the upper left/right and lower right quadrants had less than 1% of events. FMO-controls were also used. Intracellular stains were performed on paraformaldehyde fixed cells using eBioscience permeabilization buffer.

### Western blot

Cells were treated with lysis buffer (1% Triton X-100, 150 mM NaCl, 20 mM Tris, pH 7.5, 2 mM EDTA, 1 μg/ml each of aprotinin and leupeptin, 0.4 mM PMSF, 0.4 mM sodium-ortho-vanadate, 0.5 mM DTT) for 10 minutes at room temperature then insoluble debris was pelleted. CD40, CD4, CD3, and TCRβ levels were analyzed by western blot, 10 μg protein/lane [24]. As internal loading standard, membranes were stripped and stained with Coomassie Blue R-250 and a representative band is shown. Data was acquired using a PXi4 imaging system (Synoptics Inc., Frederick, MD, USA).

### Cytokine assays

Lymphocytes were cultured in the absence/presence of CD3-, CD28-, and CD40-crosslinking (biotinylated antibodies followed by streptavidin; CD3 (145-2C11) at 1 μg/ml, CD28 (37.51) at 5 μg/ml, CD40 (1C10) at 10 μg/ml, and streptavidin at 1 μg/ml) for 5 days. Supernatant was harvested and assayed for cytokines using a FlowCytomix^™^ kit from eBioscience, Inc. (San Diego, CA, USA; BMS822FF). Alternatively, cells were cultured for 3 days with the stimuli, then Brefeldin A was added during the last two hours of culture. The cells were then stained for CD4 and CD40 followed by intracellular stain for cytokines.

### Immunohistochemistry and histology

For histology, animals were perfused blood free with PBS then tissues were harvested and either frozen (for CD3 immunohistochemistry) or fixed and paraffin embedded (for luxol fast blue staining). Immunohistochemistry for CD3 and sectioning for Luxol Fast Blue staining was performed by the histology core at the Department of Pathology, School of Medicine, University of Colorado Denver. Luxol Fast Blue staining was done using a kit from ScyTek Laboratories (Logan, UT, USA). To ensure that no observed differences in Luxol Fast Blue staining were due to differential staining times between samples, control and EAE samples were stained together in batch and only compared to the samples within the batch.

### Statistical analysis

Data analysis was performed using GraphPad Prism from GraphPad Software, Inc. (La Jolla, CA, USA) employing tests as indicated in the text/figure legends.

## Results

### EAE induction expands Th40 cells, which infiltrate brain and spinal cord

**Fig 1:**
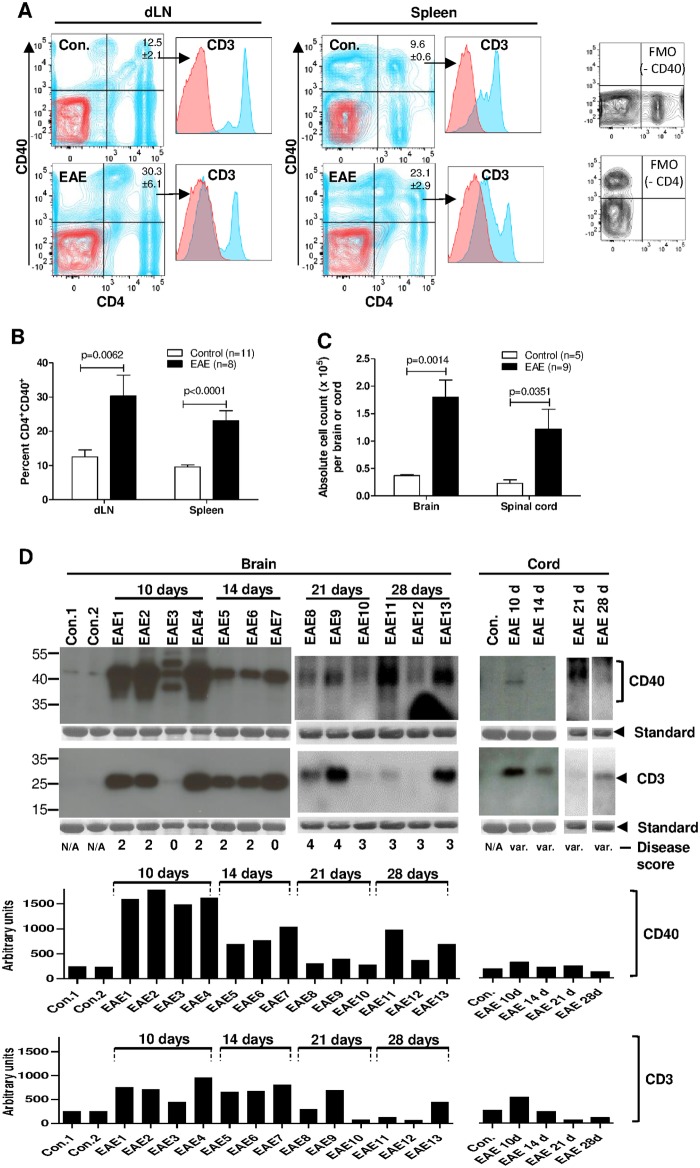
EAE induction causes expansion of the Th40 cell subset and infiltration of Th40 cells into brain and spinal cord tissues. Female C57BL/6 mice were EAE induced. (**A**) Representative plots of CD4 and CD40 stained cells (blue) in peripheral draining lymph nodes (dLN) and spleens from age- and sex-matched, wild type, control (without injection of any EAE induction component) and EAE induced mice (disease score of at least 2). Gate was set from isotype (red) and FMO (separate plots) controls. CD3 expression in CD4^+^CD40^+^ quadrant was confirmed (histograms). (**B**) Graph depicting percent Th40 cells from cumulative data in B (Control, n = 11; EAE, n = 8). Data are represented as mean ± SEM. Significance was calculated by two-tailed t-test. (**C**) Graph depicting absolute number of T cells recovered from brains and spinal cords of EAE (n = 9; disease score of at least 2) and control (n = 5) mice. Cells were MHCII and CD11b depleted to remove B cells, macrophages and contaminating microglial cells. Data is represented as mean +/- SEM. Significance was calculated using one-tailed t-test. (**D**) CD40 and CD3 western blots on T cells recovered from brains and spinal cords of control and EAE induced mice as in D. Disease scores are noted below each lane. Control samples from both brain and spinal cord were pooled from 3 mice to achieve enough cells for western blot. EAE samples from spinal cord were pooled from 3 mice 10 (10 d), 14 (14 d), 21 (21 d), and 28 (28 d) days post induction. As an internal control, membranes were stripped and stained with Coomassie blue and a representative band is shown below each blot (Standard). Graphs depict densitometry for CD40 and CD3. It should be noted that the exposure times in the western blots are different between brain and spinal cord samples in order to better visualize spinal cord samples. However, densitometry was done on the same exposures for brain and spinal cord. Data in figure represent at least 3 experiments.

C57BL/6 mice do not spontaneously develop autoimmune disease, however, EAE is inducible and requires autoimmune components. To evaluate Th40 cell involvement, EAE was induced in C57BL/6 mice (S1 Fig; average daily score). When mice reached a disease score of at least 2, a significant increase in Th40 was detected in both spleens (p<0.0001) and draining lymph nodes (dLN; superficial+deep cervical; p = 0.0062) when compared to un-induced control mice (Fig 1A and 1B).

We purified T cells from brains and spinal cords by first isolating mononuclear cells, then depleting MHCII and CD11b to remove B-cells, macrophages, and contaminating microglial cells (S2 Fig). In brain, there was a significantly higher number of infiltrating T cells than in samples from control animals (Fig 1C; p = 0.0014). Likewise, more infiltrating T cells were recovered from spinal cord from EAE than from control mice (Fig 1C; p = 0.0351). In both brain and spinal cord from EAE mice, the infiltrating cells expressed CD3, confirming T cells, while in control samples there was little CD3 expression (Fig 1D; bottom panel). When assessing CD40, high expression was found in T cells from EAE mice while T cells from control mice expressed little to no CD40 (Fig 1D; top panel). CD40 expression in T cells from EAE brains was higher at 10 days than at 14 days post-EAE-induction and remained elevated compared to control at 21 and 28 days (Fig 1D; top panel). In T cells from spinal cord, CD40 was detected at 10 days but not 14 days post-EAE-induction and was detectable again at 21 days (Fig 1D; top right panels). In control spinal cord there was no CD40 expression. CD40 is known to be composed of different glycoforms [23, 25] and, while not determined here, there are 3 bands corresponding to CD40 present in T cells from the brain of EAE induced mice (Fig 1D). It is also interesting that 10 days post-EAE-induction there were 3 bands present while at 14, 21, and 28 days only one band was detectable (Fig 1D), suggesting differences in the CD40 protein itself at these time points. Additionally, EAE sample number 3, with a disease score of 0 at 10 days post-EAE-induction (Fig 1D; EAE3), displayed a lower level of both CD40 and CD3 expression than other EAE samples with higher disease scores. Finally, densitometry revealed that expression of both CD40 and CD3 was higher at earlier time points, 10 and 14 days, than at later ones, 21 and 28 days (Fig 1D; graphs).

### T cell CD40 signaling promotes Th1 and Th17 cytokines

**Fig 2:**
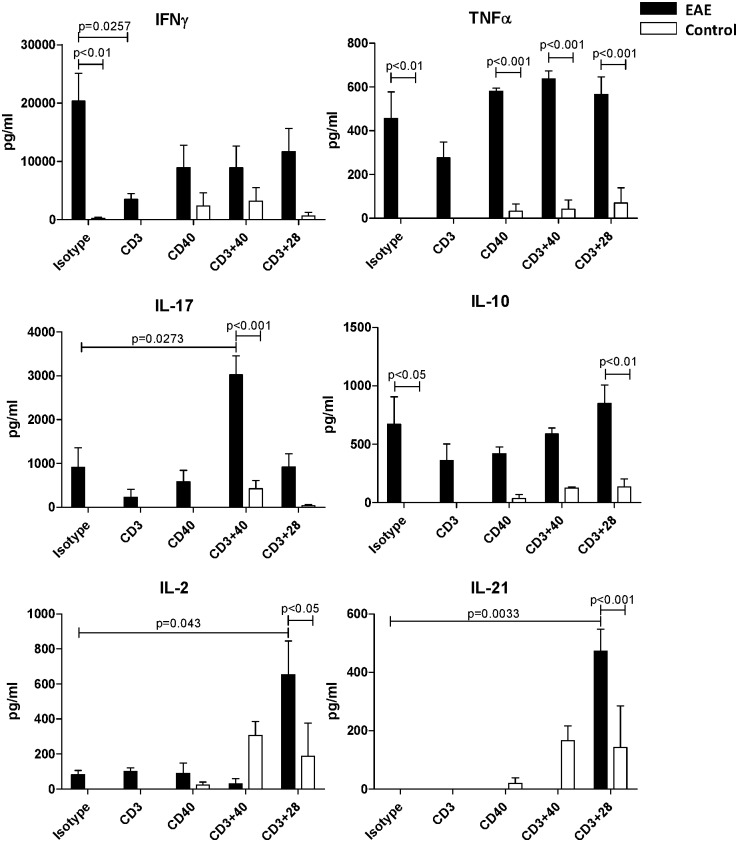
Draining LN lymphocytes from EAE mice secrete high levels of IFNγ. dLN were isolated from control (n = 2) and EAE induced mice (10 days post-EAE-induction; n = 3; EAE scores 2, 2, and 0) and lymphocytes purified. Lymphocytes were cultured in the absence/presence of CD3, CD28, and CD40 stimulation for 5 days. Cytokines in the supernatant were assayed. There were significant differences in cytokine production between lymphocytes from EAE and control mice (Two-way ANOVA) as well as between stimulated and isotype treated EAE lymphocytes (two-tailed t-test). Data represent 2 experiments.

To assess cytokine production by T cells in EAE, dLN were harvested from EAE induced and control mice 10 days after EAE induction. Given that severe EAE was induced in CD28^-/-^ mice but was controlled by blocking CD40 signals [15], we considered that CD40 can be co-stimulatory on T cells [26, 27]. Purified lymphocytes were cultured with and without CD3, CD28, and CD40 stimulation, then secreted cytokines were assayed. *In-vitro* isotype treated lymphocytes from EAE mice produced IFNγ, TNFα, IL-17, and paradoxically, IL-10, while lymphocytes from control mice did not (Fig 2). This likely is because the cells from EAE induced mice are already activated *in-vivo* since the disease course is already well on the way by day 10. By far, the highest cytokine level produced in EAE was IFNγ, which was at 20 ng/ml while the other cytokines were at 1 ng/ml or lower. In lymphocytes from EAE mice, CD3 stimulation alone significantly decreased production of IFNγ compared to isotype treated cells (Fig 2; p = 0.0257; t-test). CD40 and CD28 co-stimulation counteracted some of the CD3-alone effect (Fig 2). While not significantly different, a similar effect was seen in TNFα production. CD3+CD40 stimulation of lymphocytes from EAE mice tripled the expression of IL-17 compared to unstimulated cells (Fig 2; p = 0.0273; t-test) while CD3+CD28 stimulation induced significant expression of both IL-2 and IL-21 (Fig 2; p = 0.0430 and p = 0.0033 respectively; t-test). In cells from untreated controls, CD40 co-stimulation induced moderate levels of IFNγ, lower levels of IL-17, and higher levels of IL-2 and IL-21 (Fig 2). In these naïve lymphocytes from control mice, CD3 stimulation alone did not induce any cytokine production.

### CFA drives the expansion and migration of Th40 cells

**Fig 3:**
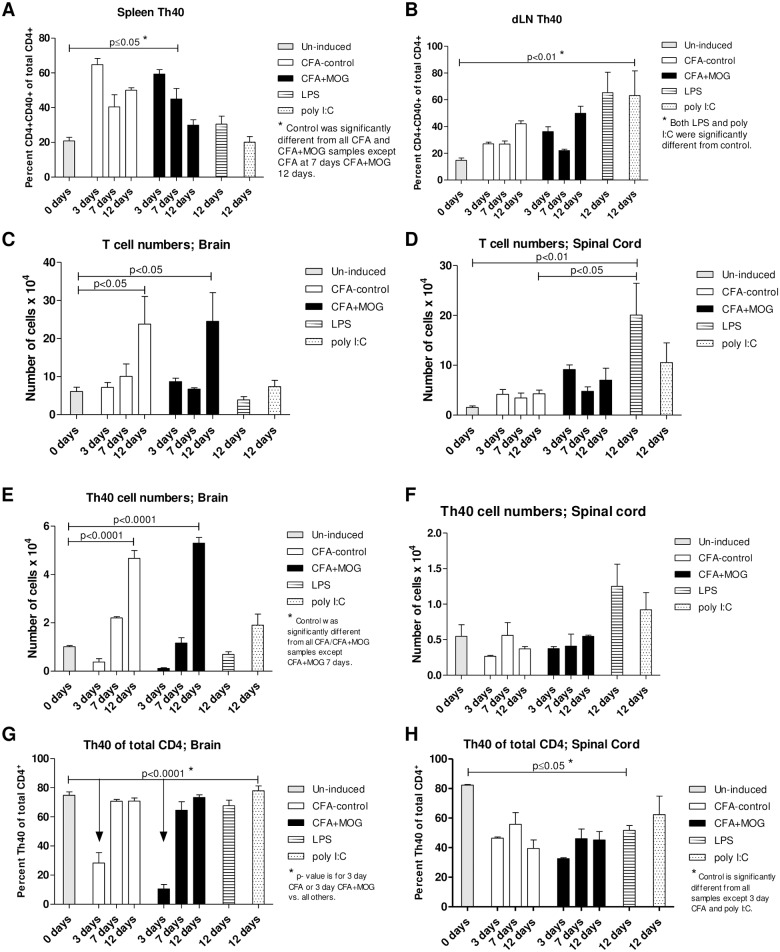
CFA induces expansion of Th40 cells. Mice were challenged with the EAE induction regimen with (CFA+MOG) or without (CFA-contol) MOG_35-55_. Alternatively, mice were challenged with LPS or poly I:C. After 3, 7, and 12 days (n = 3 per time point in each group; disease scores for all mice was 0, except in the 3 CFA+MOG mice at 12 days where the scores were 2, 3, and 4 respectively) lymphocytes were purified from (**A**) spleens and (**B**) dLN and stained for CD3, CD4, and CD40 for flow cytometry. Cells were first gated on CD3 then CD4 and CD40 levels were assessed in that gate. All gates were set from isotype/FMO controls. T cells were recovered from (**C**) brains and (**D**) spinal cords and enumerated. T cells from (**E**) brains and (**F**) spinal cords were stained and gated as above and absolute numbers of Th40 cells calculated. Percent Th40 cells of total CD4^+^ T cells was assessed in (**G**) brains and (**H**) spinal cords. Statistical differences were calculated by One-Way ANOVA with Bonferroni post-test. Data in figure represent at least 3 experiments.

The EAE induction regimen includes CFA as an adjuvant that is constituted of heat-killed *Mycobacterium Tuberculosis H37RA* total protein suspended in mineral oil. Therefore, these foreign proteins could serve as antigens to expand T cells. Injections of mice with the EAE induction regimen in the absence of MOG_35-55_ does not induce EAE symptoms [2, 28], however, expansion of Th40 cells in spleen occurred as much with CFA-control as with CFA+MOG_35-55_ compared to spleens from un-induced mice (Fig 3A; all samples except CFA 7 days and CFA+MOG_35-55_ 12 days were significantly different from control, p≤0.05). Comparing CFA-control and CFA+MOG_35-55_ induced Th40 cell expansion in spleen, no differences were observed at any time point (Fig 3A). Lipopolysaccharide (LPS; a gram-negative bacterial membrane protein) and polyinosinic:polycytidylic acid (poly I:C; representing double stranded viral RNA) are commonly used in animal models to simulate bacterial and viral infections, respectively. LPS is a TLR4 agonist [29] while poly I:C is a TLR3 agonist [30], activating innate immune cells, antigen presenting cells, and T cells. Neither LPS nor poly I:C caused a significant expansion of Th40 cells in spleens of injected mice (Fig 3A). While not significant when considering all the different treatments together, in dLN, there was a significant increase in Th40 cell levels in response to CFA-control at 12 days (p<0.0001) and CFA+ MOG_35-55_ at 3 (p<0.001) and 12 days (p<0.0001) compared to un-induced mice if LPS and poly I:C were not taken into consideration (Fig 3B; One-Way ANOVA performed separately from LPS and poly I:C). No difference was observed in dLN, at any time point, between CFA-control and CFA+MOG_35-55_ (Fig 3B). Interestingly, LPS or poly I:C caused an expansion of Th40 cells in dLN compared to un-induced mice (Fig 3B).

In separate work, we demonstrated that physical trauma is sufficient to induce T cell transmigration into the brain [31]. Therefore, we determined whether another type of insult, CFA-control injection, could also cause this to occur. T cells were purified from CNS of un-induced and induced mice (S2 Fig) and enumerated. It was evident that, over time, there was an increase in the number of T cells in the brain from both CFA-control and CFA+MOG_35-55_ induced mice compared to un-induced mice, but there was no difference between CFA-control and CFA+MOG_35-55_ challenged mice (Fig 3C). As in dLN, while not significant when considering all the different treatments together, in spinal cord, there was a significant increase in T cell levels in response to CFA-control at 12 days (p<0.0001) and CFA+ MOG_35-55_ at 3 (p<0.001) and 12 days (p<0.0001) compared to un-induced mice if LPS and poly I:C were not taken into consideration (Fig 3D; One-Way ANOVA performed separately from LPS and poly I:C). LPS, also caused an influx of T cells into the spinal cord at 12 days post-injection (Fig 3D). When actual Th40 cell numbers were specifically considered, there was a significant increase in infiltrating Th40 cells in the brain in response to both CFA-only and CFA+ MOG_35-55_ at 12 days (Fig 3E). Th40 cell numbers in the spinal cord were not significantly different from un-induced mice at any time point with any treatment (Fig 3F). Th40 cell percentage of total CD4 T cells in the brain decreased at the 3-day time points for both CFA-control and CFA+MOG_35-55_ induced mice but was no longer different at the later time points (Fig 3G). In spinal cord, the Th40 cell percentage decreased in response to all treatments except CFA-only at 7 days and poly I:C (Fig 3H). When total CD4 and CD8 T cell contents of brain and spinal cord were considered, similar results to those of Th40 cells were found (S3 Fig).

### CFA drives activation and cytokine production in Th40 cells

**Fig 4:**
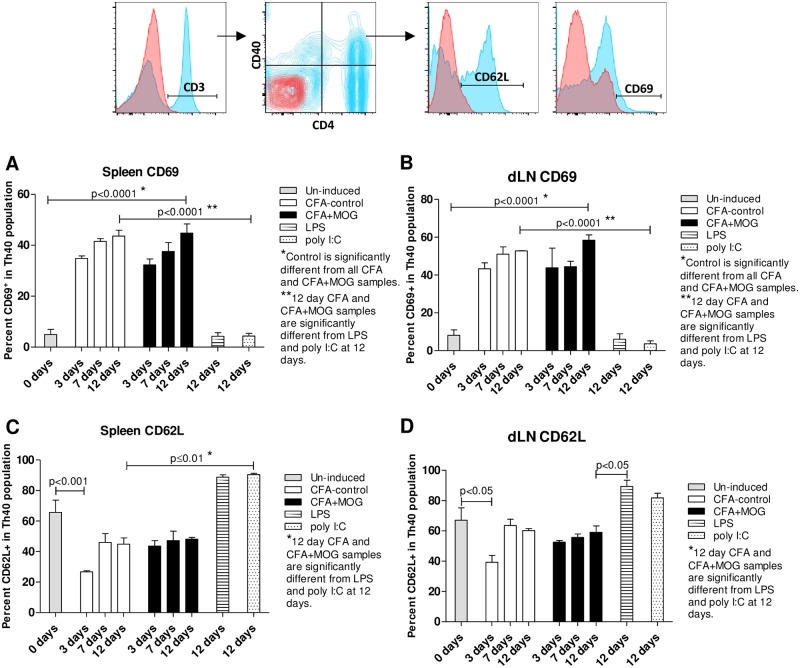
CFA alters CD69 and CD62L expression on Th40 cells in EAE. Mice were challenged with the EAE induction regimen with (CFA+MOG) or without (CFA-control) MOG_35-55_. Alternatively, mice were challenged with LPS or poly I:C for 12 days or were left completely untreated (Un-induced). After 3, 7, and 12 days (n = 3 per time point in each group; disease scores for all mice was 0, except in the 3 CFA+MOG mice at 12 days where the scores were 2, 3, and 4 respectively) lymphocytes were purified from spleens and dLN and stained for CD4, CD40, CD69, and CD62L. All gates were set from isotype and FMO controls. CD69 and CD62L expression in Th40 cells was assessed in spleen (**A** and **C** respectively) and dLN (**B** and **D** respectively). Statistical differences were calculated by One-Way ANOVA with Bonferroni post-test. Data in figure are representative of 3 experiments.

Increased CD69 expression is associated with activated T cells and CD62L is associated with retention of T cells in lymph nodes. Both CFA-control and CFA+MOG_35-55_ caused an increase in CD69 expression in splenic Th40 cells compared to Th40 cells from un-induced mice, but CFA-control and CFA+MOG_35-55_ were not different from each other (Fig 4A). LPS or poly I:C had no impact on Th40 cell CD69 expression in spleen. Similar to the spleen, in dLN there was an increase in Th40 cell CD69 expression with both CFA-control and CFA+MOG_35-55_ compared to Th40 cells from un-induced mice (Fig 4B). CD62L expression was decreased in splenic Th40 cells from CFA-control treated mice at 3 days compared to un-induced mice but that difference was no longer apparent at later time points (Fig 4C). Likewise in dLN, there was a decrease in CD62L expression when comparing Th40 cells from CFA-control mice at 3 days to Th40 cells from un-induced mice but that difference was not evident at later time points (Fig 4D). LPS and poly I:C had no impact on Th40 cell CD62L expression compared to un-induced mice but were different from the 12-day time points in both CFA-control and/or CFA+ MOG_35-55_ Th40 cells (Fig 4C and 4D). When the total CD4 population was considered, CD69 and CD62L expression results very similar to those in the Th40 population were found (S4 Fig). Additionally, central and effector memory cells were increased in symptomatic EAE mice compared to un-induced control mice (S5 Fig).

**Fig 5:**
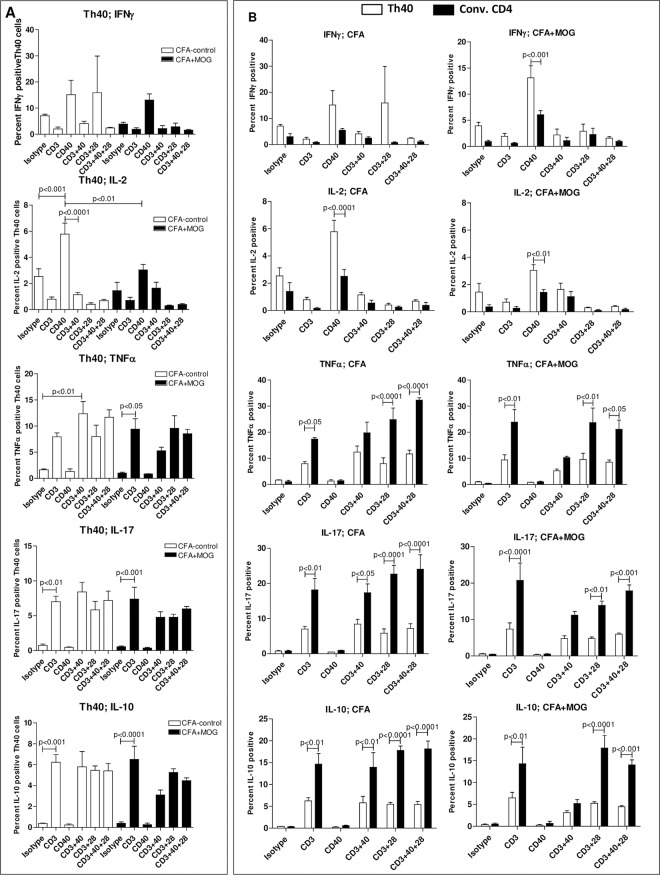
T cells from CFA-control challenged mice have a similar cytokine production potential compared to those from CFA+MOG_35-55_ challenged mice. dLN lymphocytes were purified from CFA-control and CFA+MOG challenged mice, 12 days after challenge, then the cells were cultured in the absence/presence of CD3, CD28, and/or CD40 stimulation or cultured with isotype antibodies for 3 days. Brefeldin A was added the last 2 hours, then the cells were stained for CD3, CD4, CD40, as well as for different cytokines (intracellularly). Cells were gated on CD4^+^CD40^+^ (Th40) and CD4^+^CD40^-^ (Conv. CD4; CD3 expression was confirmed in both populations) then cytokine levels were assessed. Gates were set from isotype controls. (**A**) Graphs depicting cytokine production by Th40 cells from CFA-control and Th40 cells from CFA+MOG challenged mice. (**B**) Graphs comparing cytokine production by Th40 cells to cytokine production by conventional CD4 T cells within either CFA-control (left) or CFA+MOG (right) generated cells. Statistical differences in A were calculated by One-Way ANOVA with Bonferroni post-test. Statistical differences in B were calculated by Two-Way ANOVA. Data in figure are representative of 3 experiments.

Th40 cells were isolated from dLN 12 days after CFA-control or CFA+MOG_35-55_ challenge and were then cultured in the absence/presence of CD3, CD28 and/or CD40 stimulation followed by intracellular staining for cytokines. While not measuring the cytokine secretion of cytokines, this measures the capacity to produce cytokines. While not reaching significance in all cases, CD3 stimulation induced increased expression of TNFα, IL-17, and IL-10 in Th40 cells, while CD40 stimulation induced increases in IFNγ and IL-2 in those cells (Fig 5A). In the case of CD40 stimulated increase of IFNγ and IL-2, the increase was abrogated if CD3 was stimulated simultaneously (Fig 5A). There were few noted differences between stimulated Th40 cells from CFA-control and Th40 cells from CFA+MOG_35-55_ induced mice (Fig 5A; significant differences noted in the figure). When comparing Th40 cells to conventional CD4 T cells within either CFA-control or CFA+MOG_35-55_ generated cells, many significant differences were noted between the two cell subsets, however, there were again few differences between cells generated from CFA-control and CFA+MOG_35-55_ induced mice (Fig 5B). Most notably, Th40 cells from CFA+MOG_35-55_ induced mice, but not from CFA-control induced mice, produced significantly more IFNγ in response to CD40 stimulation than conventional CD4 T cells from the same mice (Fig 5B). Th40 cells from both CFA-control and CFA+MOG_35-55_ induced mice produced more IL-2 in response to CD40 stimulation than conventional CD4 T cells from the same mice (Fig 5B). CD3-alone stimulation increased TNFα, IL-17, and IL-10 production by both Th40 and conventional CD4 T cells derived from both CFA-control and CFA+MOG mice, however, production of those cytokines was consistently higher in the conventional CD4 T cells (Fig 5B). Co-stimulation with CD40 and/or CD28 in addition to CD3 did not induce any increase in the production of TNFα, IL-17, or IL-10 (Fig 5B).

### The presence of MOG_35-55_ shapes the composition of the TCR repertoire in Th40 cells

**Fig 6:**
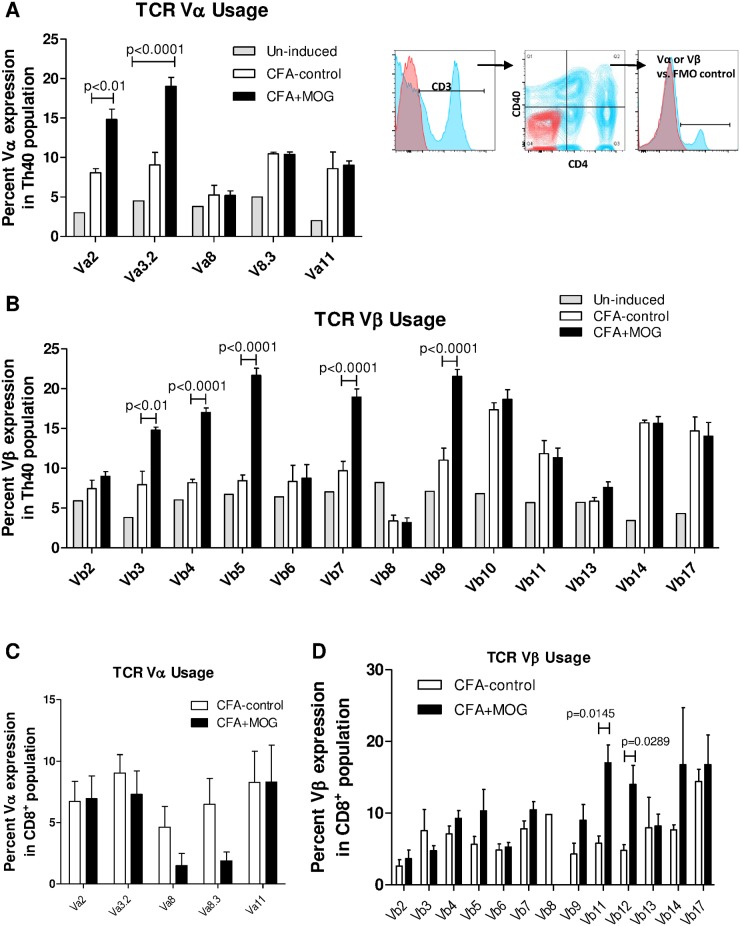
MOG_35-55_ shapes the TCR expression profile. Mice were challenged with the full EAE regimen with (CFA+MOG) or without (CFA-control) MOG_35-55_ or were not challenged at all (Un-induced). After 12 days, lymphocytes were purified from dLN and stained for CD4, CD8, CD40, and TCR Vα, or TCR Vβ individually as indicated. Representative histograms/dot plots are shown (Red, isotype control; Blue, stained sample (CD3, CD4, CD40, or Vα/β)). Mean percent expression ±SEM in Th40 cells (**A** and **B**) and in CD8 T cells (**C** and **D**) is shown. All gates were set from isotype controls. Statistical difference was calculated by Two-Way ANOVA (A and B) or two-tailed t-test (D). Data in figure are representative of 3 experiments.

From the above data it appears that CFA is driving the Th40 cell expansion as well as many of the cellular functions in those cells, yet CFA does not precipitate disease symptoms [28]. Therefore, we hypothesized that there would be a difference in TCR expression between CFA-control and CFA+MOG_35-55_ challenged mice, which in turn would cause a difference in directing the Th40 cells to the target antigen within the microenvironment. dLN cells were stained for CD3, CD4, CD40, and TCR Vα and Vβ molecules. Gating on CD3^+^ then CD4^+^CD40^+^, levels of TCR molecules were assessed in the Th40 population. TCR Vα2 and Vα3.2 were significantly expanded in CFA+MOG_35-55_ challenged mice compared to CFA-control (Fig 6A; p-values in figure; 2-Way ANOVA). Likewise, five different Vβ molecules were significantly increased on Th40 cells from CFA+MOG_35-55_ challenged mice compared to CFA-control (Fig 6B; Vβ’s 3, 4, 5, 7, and 9; p-values in figure; 2-Way ANOVA). When gating on CD8 T cells, no discernable differences were noted in Vα molecule expression and only Vβ11 and Vβ12 demonstrated a significantly increased expression in CFA+MOG_35-55_ challenged mice compared to CFA-control (Fig 6C and 6D).

### Th40 cells rapidly transfer severe disease with only the need for CFA in the recipient mouse

**Fig 7:**
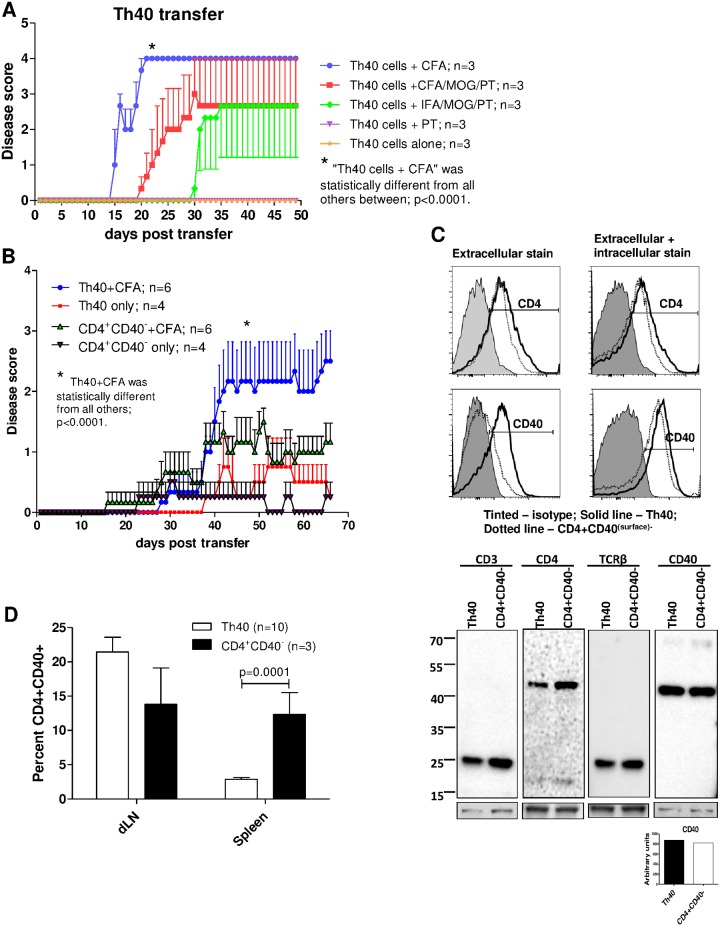
Th40 cells transfer disease. Cells, 6 x 10^7^, were transferred to C57BL/6.scid mice. (**A**) Graph depicting cumulative disease scores from Th40 cell recipients (n = 3 in each group) that received either CFA-control, full EAE regimen (CFA/MOG/PT), IFA/MOG/PT, PT alone, or nothing (Th40 cells alone). Statistical differences were calculated by One-Way ANOVA with Bonferroni’s multiple comparison test. (**B**) Graph depicting cumulative disease scores for further purified Th40 cell (further depleted on CD11b, CD11c, and CD19) or CD4^+^CD40^-^ T cell (depleted of Treg) recipients with and without CFA injection. Statistical differences were calculated by One-Way ANOVA with Bonferroni’s multiple comparison test. (**C**) Flow cytometry histograms of donor cells, prior to transfer, stained for CD4 and CD40 extracellularly (left) or extracellularly and intracellularly (right). Thick black line, Th40 cells; Dotted line, CD4^+^(CD40^(surface)-^) cells; Tinted, isotype control. Below histograms, western blot for CD3, CD4, TCRβ (H57-597), and CD40 on donor cells prior to transfer. As a loading standard, membranes were stripped and stained for coomassie blue and a representative band is shown. Graph below western blot is densitometry of the CD40 band divided by the loading standard. (**D**) Cells recovered from spleen and dLN from Th40 or CD4^+^(CD40^(surface)-^) T cell recipient mice were stained for CD3, CD4, and CD40. The CD4/CD40 gate was set from isotype controls. CD3 expression was confirmed. Graph depicts mean ± SEM percent CD4^+^CD40^(surface)+^ cells from cumulative data in each group. Statistical differences were calculated by t-test. Data in figure are representative of at least 2 experiments.

The importance of CD4 T cells as drivers in the development of EAE is clear [4, 5, 32]. Disease transfer experiments in the C57BL/6 EAE model are typically done by inducing donor mice with the EAE-regimen then culturing the donor cells in the presence of additional specific antigen and IL-12 prior to transferring them into recipients [9]. Alternatively, sorted CD4 donor T cells can be transferred to scid-recipients followed by administration of the full EAE-regimen [10], or MOG-reactive TCR transgenic T cells can be used without the need for further culture or recipient treatment [33]. We transferred primary Th40 cells to scid recipient mice followed by a subcutaneous injection of CFA. There was no need for *in-vitro* culturing or injection of the EAE-regimen; CFA-control, without MOG_35-55_ and PT, precipitated severe disease where the symptoms progressed very quickly and the mice succumbed to disease overnight once a level 4 was reached (Fig 7A). In fact, when the full EAE-induction regimen was administered to recipient mice, disease onset was delayed and not all mice became symptomatic (Fig 7A). In a group where Th40 cells were transferred and MOG_35-55_ was administered in incomplete Freund’s adjuvant (IFA) followed by PT, disease onset was delayed and not all mice became symptomatic (Fig 7A).

### Th40 cells transfer disease with greater severity than conventional CD4^+^CD40^-^ T cells

Since Th40 cells transferred disease, we assessed whether *in-vivo* antigen primed Th40 cells were more efficient than conventional CD4^+^(CD40^(surface)-^) T cells. To further ensure that the Th40 cells were devoid of B-cells, macrophages, and dendritic cells we further purified them by removing CD11b, CD11c, and CD19. Conventional CD4^+^(CD40^(surface)-^) T cells were Treg depleted. While the disease course was slower than in Fig 7A, the further purified Th40 cells transferred more severe disease than conventional, Treg depleted, CD4^+^(CD40^(surface)-^) T cells (Fig 7B; p< 0.01; One-way ANOVA). Interestingly, when following the mice for a longer time, donor Th40 cells, without accompanying CFA, transferred disease but a milder form was observed (Fig 7B). As the conventional CD4^+^(CD40^(surface)-^) T cell recipient mice demonstrated EAE symptoms, we determined whether the donor cells were completely CD40^-^ or whether CD40 had been induced. Prior to transfer, sorted CD4^+^(CD40^(surface)-^) cells were confirmed to be negative for cell surface-CD40 expression (Fig 7C; histograms). However, when stained intracellularly, it was evident that the CD4^+^CD40^(surface)-^ cells harbored CD40 protein. Western blot of whole cell extracts from donor cells prior to transfer, likewise, revealed an intracellular level of CD40 similar to that of Th40 cells (Fig 7C). We confirmed the T cell nature of both populations by western blots for CD3, CD4 and TCRβ (Fig 7C). Post-transfer recovered T cells from spleens and lymph nodes of CD4^+^CD40^(surface)-^ recipient mice that had reached a disease score of at least 2 demonstrated the presence of newly induced surface-CD40^+^ CD4^+^ T cells (Fig 7D). This indicates that the disease process caused a conversion of the transferred cells to a Th40 phenotype. In addition, we recovered T cells from the brains and spinal cords of the recipient mice and found no difference in the amount of infiltrating T cells between Th40 and conventional CD4^+^(CD40^(surface)-^) T cell recipients (S6 Fig). This indicates that a difference in number of infiltrating T cells cannot account for the difference in disease severity, rather a phenotypic, qualitative difference is in play.

### Th40 cells infiltrate both brain and spinal cord in EAE disease transfer mice

**Fig 8:**
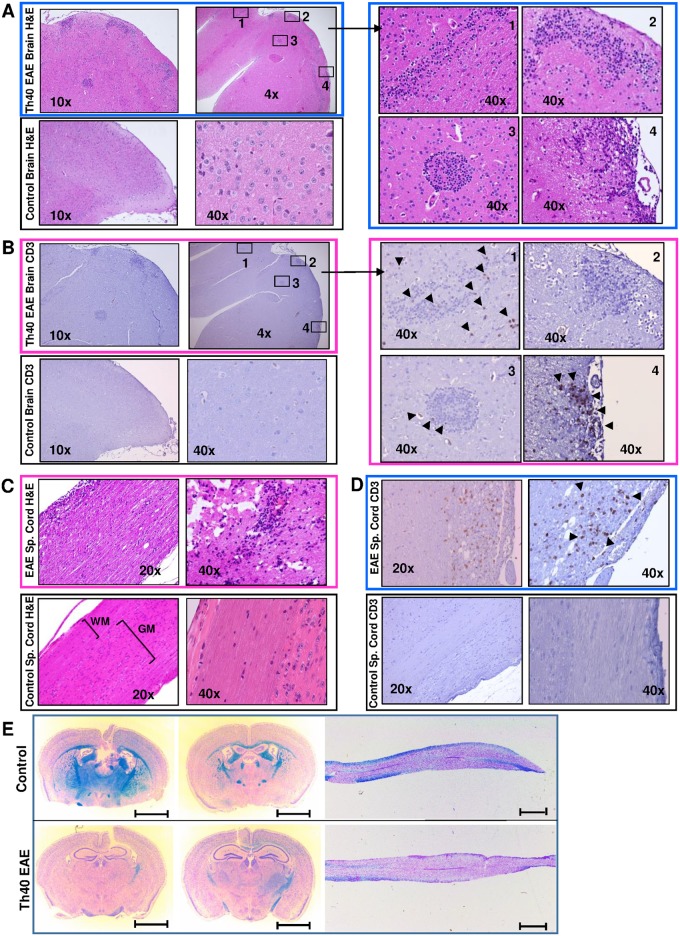
Th40 cells infiltrate brain and spinal cord in EAE disease transfer mice with resulting demyelination. Splenic Th40 cells from EAE induced C57BL/6 mice were transferred to C57BL/6.scid mice. Brain and spinal cord sections from Th40 cell recipient and un-induced control mice are shown. (**A**) H&E stained Th40 EAE (at disease score 4) and control brain (**B**) CD3 immunohistochemistry on Th40 EAE (at disease score 4) and control brain. In both A and B numbered squares in the top left section are enlarged (40x; right panels). Arrow heads indicate CD3 positive areas. (**C**) H&E stained Th40 EAE (at disease score 4) and control spinal cord. (**D**) CD3 immunohistochemistry on Th40 EAE (at disease score 4) and control spinal cord (white matter, WM, and grey matter, GM, are indicated). (**E**) Representative Luxol Fast Blue stained coronal brain and longitudinal spinal cord (mid-thoracic to tail) sections from Th40 EAE (bottom panels; n = 3) compared to control mice (top panels; n = 2). Scale bars are 2 mm. Data in figure are representative of at least 3 experiments.

In histological samples of brain from Th40 cell recipient mice, potential lesions were observed mainly in the cortex while a comprehensive examination of brains from un-induced control mice demonstrated no lesions (Fig 8A). As the recipient mice (C57BL/6.scid) lack T and B cells, any T cells that infiltrate must originate from the transfer. However, other immune cells from the recipient mouse could infiltrate the brain; therefore, we performed immunohistochemistry for CD3. In the EAE brain, many lesions contained infiltrating CD3^+^ cells (Fig 8B; areas 1, 3, and 4) while some contained no CD3^+^ cells (Fig 8B; area 2). In some cases, there were areas outside of the visible lesion that contained CD3^+^ cells (Fig 8B; area 1). The control brain did not demonstrate CD3 stain (Fig 8B). H&E staining of the spinal cord from symptomatic Th40 cell recipient mice revealed infiltrating cells throughout the spinal cord with a higher concentration in the white matter (Fig 8C) and accompanying loss of striation. CD3 staining showed a more uniform pattern than found in brain (Fig 8D). As in the brain, control sections did not demonstrate CD3 stain or loss of striation (Fig 8C and 8D). Luxol Fast Blue staining of brain and spinal cord sections demonstrated wide spread demyelination in both tissues from Th40 cell recipient mice compared to un-induced control mice (Fig 8E; loss of blue staining myelin).

## Discussion

The etiology of MS remains unclear but unquestionably autoimmune components play a major role, which became understood from the EAE mouse model. This model demonstrated the involvement of several immune cells, many of which have been identified in human MS lesions, with T cells taking center stage as culprits. Miller et al. outlined two phases of actively induced EAE; the induction phase and the effector phase. The induction phase involves priming of antigen-specific CD4^+^ T cells while the effector phase consists of: [1] migration of activated antigen-specific T cells to the CNS; [2] elaboration of cytokines and chemokines by those antigen-specific T cells to induce influx of peripheral mononuclear phagocytes into the CNS; [3] activation of infiltrating monocytes/macrophages as well as CNS-resident microglial cells by T cell derived cytokines; and [4)] demyelination of CNS axonal tracts [9].

We can now adjust the disease phase paradigm so that the induction phase includes a T cell expansion that is independent of CNS antigens. We discovered that CD4^+^CD40^+^ T cells, Th40 cells, become expanded in EAE-induced mice. In this model, Th40 cell expansion relies only on the CFA component of the EAE induction regimen, as does the cytokine expression phenotype of those Th40 cells. However, MOG_35-55_ antigen, in addition to CFA, is necessary to elicit disease. MOG_35-55_ induces a somewhat altered cytokine production potential but foremost shapes an altered TCR profile. This constitutes the second part of the induction phase, i.e. priming of antigen specific T cells. Therefore, it appears that CFA is responsible for part of the induction phase and several aspects of the effector phase stages, at least with regard to the T cell activities. CFA-control caused an upregulation of CD69 and a downregulation of CD62L expression on Th40 cells in both spleen and dLN and there was no difference compared to CFA+MOG_35-55_, indicating that the CFA component is responsible for activating the Th40 population. Certainly, Th40 cells migrated into the CNS as readily with CFA as with CFA+MOG_35-55_. CFA-activated Th40 cells achieved effector status, producing cytokines to the same extent as CFA+MOG_35-55_, with few noted differences. These data suggest that in order to precipitate MS in a human subject, there is not only a need for the presence of specific antigen and T cells capable of recognizing that antigen, but there is also the necessity of a ramped up generalized inflammation that likely supports both the activity and migration of the antigen specific cells, as well as the presentation of the specific antigen to those cells. Therefore, in order to prevent or ameliorate disease, it may not be necessary to definitively target the antigen specific cells, it could be equally effective to dampen or avoid generalized inflammation by avoiding inflammation causing agents/situations such as viruses or trauma. Additionally, the results in Fig 2 demonstrate that, depending on what stimuli the activated T cells encounter, they will either secrete inflammatory or regulatory cytokines, i.e. CD40 signals induced secretion of inflammatory cytokines while CD28 induced secretion of regulatory cytokines. IL-2 promotes Tregs and knockout of IL-2 or IL-2-receptor causes lethal autoimmunity [34], therefore CD28 drives a more regulatory environment while CD40 promotes an inflammatory environment.

IL-21 expression is, in some cases, reported to be associated with MS, while in other cases it is reported to have no association. Likewise, in the EAE mouse model, its role remains controversial [35]. It is curious that CD3+CD40 stimulation induced IL-21 production by the T cells from un-induced control mice while cells from EAE-induced mice produced this cytokine in response to CD3+CD28. These data do not bring clarity to the IL-21 contribution in EAE but it is likely that the discrepancies regarding this cytokine stem from many issues such as when in the disease process, by which cell, in response to what stimulus etc. was IL-21 measured and, perhaps most importantly, what other cytokines were present and at what level?

LPS and poly I:C are often used to simulate bacterial and viral infections. Therefore, we assessed whether these agents could cause an expansion of Th40 cells. Interestingly, those agents had some effect on Th40 cell expansion and tissue infiltration but not to the extent of CFA. This likely is because CFA contains heat killed M. Tuberculosis, i.e. all mycobacterial components, which may trigger many immune pathways simultaneously, while LPS and poly I:C are single, pure components. Also, in the case of poly I:C, it is a synthetic mimic, not a true, coding RNA.

Our disease transfer data support the notion of the necessity for a generalized activation of the immune system (in this case achieved by CFA) together with a target-specific honing of the TCR profile by CNS antigen (in this case MOG_35-55_) to precipitate severe disease. Many attempts have been made to associate a specific infectious event or agent with MS. Our data suggest that any strong infection leading to Th40 cell expansions will suffice in susceptible individuals. While we were able to transfer mild disease with further purified donor Th40 cells only, disease was much more severe when the recipients received CFA as well. Other disease transfer protocols use total lymph node or splenic cells from EAE donors with *in-vitro* exposure to more antigen and IL-12 [8, 9]. In those protocols all the cells, including antigen presenting cells (APC), are transferred, circumventing the need to activate the recipients’ own APC. In yet other protocols, enriched donor T cells are transferred but to precipitate disease, the full EAE regimen is required in the recipient [10], presumably to activate host APC. To our knowledge, this is the first report demonstrating adoptive transfer of EAE with primary, non-TCR transgenic T cells without the need for additional exogenous antigen. In fact, disease transfer kinetics were slowed by exogenously provided MOG_35-55_, which likely reflects that the antigen specific cells became occupied responding to this source of antigen before attacking endogenous sources. If IFA together with MOG_35-55_ was given, disease kinetics slowed, again demonstrating that once the antigen specific T cells are present they only need the support of a generalized immune activation, not the presence of more exogenous antigen.

In C57BL/6 mice, both relapsing-remitting and chronic EAE can be induced [7]. When mice were given a lower amount of antigen, as well as a lower amount of CFA, the disease course was relapsing-remitting (40%) or resolved (60%) after the initial disease symptoms. When mice were given a higher amount of antigen and CFA, the disease course was chronic, indicating that while antigen dose matters, in light of our findings, clearly so does generalized inflammation. Perhaps, with the lower dose of CFA, the mice resolve the generalized inflammation that supports the activity of antigen-specific T cells, allowing recovery from disease.

Another interesting aspect of our disease transfer experiments is that when the Th40 cells were further purified by removing CD11b, CD11c, and CD19, not only MHCII, to remove as many antigen presenting cells as possible (compare Fig 7B to 7A), the disease kinetics were slowed down. However, Th40 cells transferred more severe disease than conventional, Treg depleted, CD4^+^ T cells. This further indicates that there are immune cells induced by generalized inflammation that are not completely necessary but support the activities of the culprit T cells. It will be interesting to, in the future, determine what those immune cells are and the how they support the Th40 driven disease. Do they help present antigen better or do they produce supportive cytokines etc.?

It was recently discovered that the CNS contains subdural lymphatic vessels, putting into question the immune privileged nature of the brain [36]. We recovered T cells from the brains of CFA-control treated mice and have previously demonstrated that surgery induced a rapid influx of activated T cells, primarily Th40 cells, into the brain [31]. Therefore, trauma, including surgery, or generalized inflammation (induced by CFA here) may trigger a rapid influx of activated T cells into the CNS by affecting the newly discovered extension of the lymphatic system in order to protect this most vital organ. However, as many of the infiltrating cells are activated Th40 cells, with the provision of antigen, this sets the stage for breach of self-tolerance in susceptible individuals via CD40-induced TCR alteration [37].

Based on our findings and expanding on the model proposed by Miller et al., we propose the following model: [1] Induction/expansion of potentially pathogenic T cells, Th40 cells, that achieve effector status in the periphery in response to non-specific stimulation such as trauma or systemic infection; [2] Migration of Th40 cells to the CNS occurring as a response to the trauma/systemic inflammation; [3] Honing of TCRs to achieve antigen specificity (peripherally supplied antigen in the animal induction model; supplied peripherally or locally as a consequence of trauma/systemic inflammation in MS patients). Obviously step 3 may not occur, in which case disease is never precipitated but rather the inflammation is resolved and the CNS remains healthy.

We have reported an expansion of Th40 cells in MS patients [20]. The reverse translational data here indicate that these cells are sufficient to drive development of MS with only the need for a generalized inflammation once sufficient antigen is present. Surface expression of CD40 on CD4^+^ T cells was associated with a more severe disease course. Importantly, once conventional CD4^+^(CD40^(surface)-^) T cell recipients demonstrated symptoms, many of the transferred T cells had become surface-CD40^+^, further implicating CD40 as a central player in pathogenicity. CD40 induces altered TCR expression in Th40 cells [38]. It is possible that to acquire the disease causing TCRs, surface-CD40^-^ cells first have to induce CD40 on the surface and then alter their TCRs before they are capable of targeting the CNS. This could impact the timely accumulation of antigen-specific T cells while generalized inflammation is ongoing and therefore a “window of opportunity” to cause disease is missed. Recent studies demonstrate that latent infection with γ-herpesvirus-68, the murine homologue of Epstein-Barr virus, upregulates CD40 expression, facilitating and exacerbating EAE [39, 40]. Therefore, it is possible that, in a susceptible subject with latent viral infection, the immune system is already poised and only a mild additional insult is necessary to increase the generalized inflammation with subsequent disease as a result.

While significantly higher than in controls, numbers of Th40 cells in human MS subjects, primarily relapsing-remitting MS, have a wide range [20]. It will be important to assess Th40 cell levels during relapse/remission in order to evaluate whether expanded Th40 cell numbers can serve as predictors of a relapse. Th40 cells may be drivers in human MS but, importantly, expanded numbers may serve as a risk assessment tool. It will also be important to evaluate whether different T cell populations, e.g. Th40 cells, CD4^+^CD40^-^, and CD8^+^CD40^+^ T cells, cause different symptoms, explaining different types of disease course. Such knowledge could lead to therapies tailored to the specific types of MS.

## Supporting information

S1 Data Supporting information containing data represented in the manuscript figures. (PDF)

S1 Fig Female C57BL/6 mice, ages 10–12 weeks, were EAE induced. Mice (n = 5) were monitored for clinical disease scores. Average daily scores are shown.
(PDF)

S2 Fig C57BL/6 mice that were either un-induced (Control; n = 3) or EAE induced (EAE; n = 3; taken at disease score of 2, 3, and 3) were perfused with PBS then brains were harvested. Tissue was homogenized to single cell suspension then mononuclear cells were purified on a 37%/70% discontinuous Percoll gradient. Resulting cells were washed then MHCII- and CD11b-expressing cells were depleted to remove B cells, macrophages and contaminating microglia cells. Cells were stained for surface expressed CD3, CD4, and TCRβ in flow cytometry. Gates were set based on isotype controls. Percent CD3 is from total live cells and CD4 and TCRβ are from total CD3 expressing cells.
(PDF)

S3 Fig Mice were challenged with the EAE induction regimen with (CFA+MOG) or without (CFA-contol) MOG35-55. Alternatively, mice were challenged with LPS or poly I:C. After 3, 7, and 12 days (n = 3 per time point in each group; disease scores for all mice was 0, except in the 3 CFA+MOG mice at 12 days where the scores were 2, 3, and 4 respectively) lymphocytes were purified from brains (left panels) and spinal cords (right panels) and stained for CD3, CD4, and CD40 for flow cytometry. Cells were first gated on CD3 then CD4 and CD40 levels were assessed in that gate. All gates were set from isotype controls. Percentages and numbers of total CD4 or CD8 T cells were calculated. Levels of CD40-expressing CD8 cells were determined. Statistical differences were calculated by One-Way ANOVA with Bonferroni post-test. Data in figure represent at least 3 experiments.
(PDF)

S4 Fig Mice were challenged with the EAE induction regimen with (CFA+MOG) or without (CFA-control) MOG35-55. Alternatively, mice were challenged with LPS or poly I:C for 12 days or were left completely untreated (Un-induced). After 3, 7, and 12 days (n = 3 per time point in each group; disease scores for all mice was 0, except in the 3 CFA+MOG mice at 12 days where the scores were 2, 3, and 4 respectively) lymphocytes were purified from spleens and dLN and stained for CD4, CD40, CD69, and CD62L. All gates were set from isotype and FMO controls. CD69 and CD62L expression in total CD4 T cells was assessed in spleen (A and C respectively) and dLN (B and D respectively). Statistical differences were calculated by One-Way ANOVA with Bonferroni post-test. Data in figure are representative of 3 experiments.
(PDF)

S5 Fig C57BL/6 mice were EAE induced (EAE; n = 4) or not (Control; n = 5). When the EAE induced mice reached a disease score of 2, 2, 3, and 3, respectively, spleens and dLN were harvested and stained for CD3, CD4, CD40, CD62L and CD44. Cells were gated on CD3 then central (CD62L^+^CD44^+^) and effector (CD62L^-^CD44^+^) memory cells were assessed within the total CD4 population and within the Th40 population. Gates were set based on isotype and FMO controls. Statistical difference was calculated by two-tailed t-test and significant differences are noted in the graphs.
(PDF)

S6 Fig Th40 or CD4+CD40^(surface)-^ T cells, 6 x 107, were transferred to C57BL/6.scid mice followed by CFA administration. When Th40 recipients reached a disease score of at least 2, brains and spinal cords were harvested from those mice, as well as from the CD4+CD40^(surface)-^ T cell recipients that were at lower scores at the same time points. T cells were purified and enumerated. Statistical difference was calculated by two-tailed t-test.
(PDF)
