# Supplementary material for: Th40 cells (CD4+CD40+ Tcells) drive a more severe form of Experimental Autoimmune Encephalomyelitis than conventional CD4 T cells
Source: PLoS One. 2017 Feb 13;12(2):e0172037. doi: 10.1371/journal.pone.0172037 (PMC5305068; doi:10.1371/journal.pone.0172037)
Supplement: S1 Data — (PDF) [file pone.0172037.s001.pdf]

Figure 1.

A and B

	Control											EAE							
dLN	12.00	15.80	23.00	16.00	20.70	12.10	16.60	10.90	3.24	3.18	4.12	15.50	25.00	17.50	40.80	49.10	59.30	12.20	23.00
Spleen	10.70	12.40	7.42	9.20	5.19	8.40	9.23	9.33	11.60	11.40	10.70	19.70	10.70	14.20	35.50	24.60	29.10	28.20	23.00

C

	EAE									Control				
Brain	1.000	3.500	2.800	2.400	1.140	1.780	1.620	1.360	0.620	0.425	0.300	0.400	0.340	0.380
Spinal cord	0.220	0.990	0.890	0.560	1.020	1.260	4.000	1.020	1.000	0.470	0.140	0.140	0.220	0.180

D

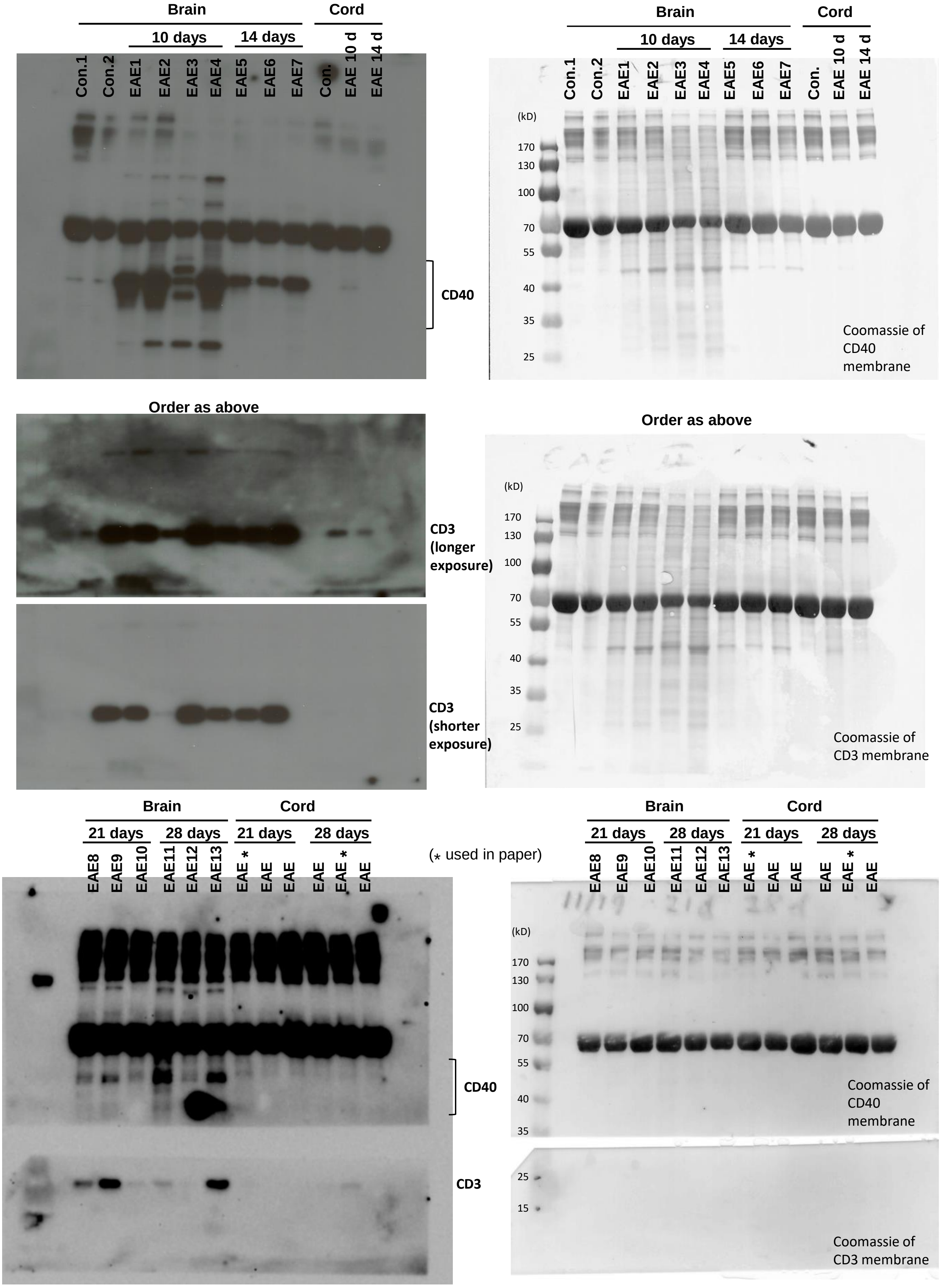

(In the 21 and 28 day westerns, membrane was cut such that CD40 and CD3 could be done separately. Therefore the Coomassie stain as control is the same for both CD40 and CD3)

Figure 2.

IFNγ

	EAE			Control	
Isotype	24856	25354	10823	420	0
anti-CD3	5076	3704	1666	0	0
anti-CD40	2418	15796	8559	4598	96
anti-CD3+40	4427	16245	6027	5504	829
anti-CD3+28	15639	15634	3758	1264	0

TNFα

	EAE			Control	
Isotype	599	555	213	0	0
anti-CD3	402	276	150	0	0
anti-CD40	577	606	561	65	0
anti-CD3+40	574	702	632	83	0
anti-CD3+28	646	646	405	139	0

IL-17

	EAE			Control	
Isotype	1619	1020	81	0	0
anti-CD3	588	96	0	0	0
anti-CD40	1101	321	321	0	0
anti-CD3+40	3878	2488	2700	611	230
anti-CD3+28	1397	374	989	64	0

IL-10

	EAE			Control	
Isotype	969	835	211	0	0
anti-CD3	338	616	125	0	0
anti-CD40	527	394	338	71	0
anti-CD3+40	687	560	516	134	115
anti-CD3+28	989	1024	538	202	66

IL-2

	EAE			Control	
Isotype	129	59	60	0	0
anti-CD3	121	119	63	0	0
anti-CD40	0	66	200	40	5
anti-CD3+40	0	0	89	386	225
anti-CD3+28	993	639	324	376	0

IL-21

	EAE			Control	
Isotype	0	0	0	0	0
anti-CD3	0	0	0	0	0
anti-CD40	0	0	0	38	0
anti-CD3+40	0	0	0	216	115
anti-CD3+28	512	578	327	285	0

Figure 3.

A

Control	CFA 3d	CFA 7d	CFA 12d	MOG 3d	MOG 7d	MOG 12d	LPS	poly I:C
22.8	57.7	33.2	47.2	55.6	40.1	35.7	39.6	17.2
16.6	69.4	33.7	51.3	64.2	37.7	24.7	25.4	16.6
23.0	67.2	54.4	51.5	58.3	57.0	29.2	26.6	26.6

B

Control	CFA 3d	CFA 7d	CFA 12d	MOG 3d	MOG 7d	MOG 12d	LPS	poly I:C
11.4	28.2	30.7	45.0	30.4	23.4	41.0	95.0	26.9
13.4	24.6	22.7	37.7	42.8	22.7	49.2	56.9	74.7
11.6	28.4	27.3	43.5	35.3	19.9	59.4	44.0	87.8
19.7								
17.9								

C

Control	CFA 3d	CFA 7d	CFA 12d	MOG 3d	MOG 7d	MOG 12d	LPS	poly I:C
11.4	28.2	30.7	45.0	30.4	23.4	41.0	95.0	26.9
13.4	24.6	22.7	37.7	42.8	22.7	49.2	56.9	74.7
11.6	28.4	27.3	43.5	35.3	19.9	59.4	44.0	87.8
19.7								
17.9								

D

Control	CFA 3d	CFA 7d	CFA 12d	MOG 3d	MOG 7d	MOG 12d	LPS	poly I:C
1.4	5.25	3.75	2.80	10.60	6.30	2.20	8.2	18.0
0.8	5.00	4.95	4.70	7.40	3.15	9.90	30.0	9.0
2.2	2.25	1.65	5.30	9.40	4.80	8.90	22.0	4.6
1.8								

E

Control	CFA 3d	CFA 7d	CFA 12d	MOG 3d	MOG 7d	MOG 12d	LPS	poly I:C
1.00	0.65	2.10	5.30	0.06	1.60	5.30	0.86	1.1
0.94	0.29	2.20	4.50	0.15	0.89	5.70	0.49	2.7
1.10	0.19	2.30	4.20	0.13	1.00	4.90	0.73	1.9

F

Control	CFA 3d	CFA 7d	CFA 12d	MOG 3d	MOG 7d	MOG 12d	LPS	poly I:C
0.99	0.26	0.27	0.37	0.33	0.24	0.58	0.65	1.40
0.20	0.25	0.52	0.43	0.36	0.58	0.54	1.70	0.63
0.53	0.29	0.89	0.31	0.43		0.52	1.40	0.73
0.47								

G

Control	CFA 3d	CFA 7d	CFA 12d	MOG 3d	MOG 7d	MOG 12d	LPS	poly I:C
72.20	42.10	71.30	66.40	5.30	73.70	73.50	62.90	73.80
75.00	24.50	68.30	73.00	16.10	53.70	76.40	75.20	84.50
81.30	18.30	72.50	72.90	10.10	66.20	70.00	64.90	75.30
64.60								
77.60								
77.90								

H

Control	CFA 3d	CFA 7d	CFA 12d	MOG 3d	MOG 7d	MOG 12d	LPS	poly I:C
83.10	45.10	52.70	49.80	33.80	39.40	52.90	53.20	48.30
81.50	46.70	70.80	30.10	31.40	52.60	48.50	56.50	51.40
83.20	47.60	44.00	38.50	32.40		34.30	45.50	87.40
81.40								

Figure 4.

A

Control	CFA 3d	CFA 7d	CFA 12d	MOG 3d	MOG 7d	MOG 12d	LPS	poly I:C
2.58	33.30	40.20	45.60	32.90	35.40	45.50	1.79	2.33
1.43	36.70	43.70	39.10	36.00	32.90	50.60	6.57	5.01
1.50	34.50	40.60	46.20	27.90	44.40	38.30	4.46	5.75
8.85								
10.60								

B

Control	CFA 3d	CFA 7d	CFA 12d	MOG 3d	MOG 7d	MOG 12d	LPS	poly I:C
2.24	45.30	58.60	52.50	32.50	44.00	57.30	1.24	6.42
4.35	37.20	45.70	53.00	34.40	39.80	54.20	5.72	1.17
4.27	47.50	48.70	52.80	64.60	49.60	63.80	11.10	3.30
13.60								
16.20								

C

Control	CFA 3d	CFA 7d	CFA 12d	MOG 3d	MOG 7d	MOG 12d	LPS	poly I:C
80.50	25.60	48.60	45.10	40.30	41.00	46.90	85.70	88.80
79.20	26.50	54.40	37.60	39.70	40.90	50.50	90.80	90.70
76.50	28.10	35.20	51.90	50.80	59.60	47.00	89.80	91.80
46.70								
45.60								

D

Control	CFA 3d	CFA 7d	CFA 12d	MOG 3d	MOG 7d	MOG 12d	LPS	poly I:C
87.60	37.30	71.50	57.80	50.40	51.60	67.50	81.40	86.70
77.10	32.70	57.80	60.50	53.40	56.00	55.30	92.60	82.50
73.50	47.90	61.40	62.30	54.00	59.40	54.20	94.20	76.20
55.20								
41.60								

Figure 5.

A; IFNγ

CFA						CFA+MOG					
Isotype	CD3	CD40	CD3+40	CD3+28	CD3+40+28	Isotype	CD3	CD40	CD3+40	CD3+28	CD3+40+28
8.02	3.25	26.10	2.69	44.00	2.86	3.570	1.470	16.200	0.027	5.630	1.430
5.98	0.82	8.71	3.79	0.89	2.15	5.230	3.030	8.560	3.890	1.600	1.190
7.27	2.05	10.60	5.73	2.84	2.19	2.990	1.270	14.600	2.640	1.460	2.120

A; IL-2

CFA						CFA+MOG					
Isotype	CD3	CD40	CD3+40	CD3+28	CD3+40+28	Isotype	CD3	CD40	CD3+40	CD3+28	CD3+40+28
3.38	0.47	7.29	0.82	0.55	0.48	0.37	0.40	3.34	0.90	0.21	0.30
1.40	0.80	5.67	1.17	0.17	0.77	2.59	1.17	2.23	2.46	0.30	0.40
2.84	1.09	4.38	1.44	0.50	0.80	1.39	0.54	3.57	1.58	0.38	0.49

A; TNFα

CFA						CFA+MOG					
Isotype	CD3	CD40	CD3+40	CD3+28	CD3+40+28	Isotype	CD3	CD40	CD3+40	CD3+28	CD3+40+28
1.72	8.27	2.23	16.00	11.80	14.50	1.06	10.50	0.87	4.82	14.30	7.83
1.37	6.55	0.60	8.03	4.20	10.50	1.26	12.20	0.75	4.43	6.38	7.61
1.84	9.06	1.13	13.10	7.98	10.10	0.70	5.57	0.92	6.62	8.05	10.20

A; IL-17

CFA						CFA+MOG					
Isotype	CD3	CD40	CD3+40	CD3+28	CD3+40+28	Isotype	CD3	CD40	CD3+40	CD3+28	CD3+40+28
1.07	5.67	0.49	10.90	7.69	6.11	0.50	9.64	0.21	3.82	4.23	5.63
0.50	6.96	0.38	6.20	3.57	5.62	0.74	8.46	0.27	4.15	5.62	5.66
0.71	8.39	0.55	8.17	6.27	9.85	0.44	4.09	0.58	6.36	4.49	6.65

A; IL-10

CFA						CFA+MOG					
Isotype	CD3	CD40	CD3+40	CD3+28	CD3+40+28	Isotype	CD3	CD40	CD3+40	CD3+28	CD3+40+28
0.31	4.88	0.17	8.61	6.27	6.29	0.20	6.02	0.57	2.23	5.91	4.81
0.46	6.40	0.43	3.60	5.27	3.98	0.64	8.88	0.12	3.34	4.71	3.94
0.37	7.41	0.11	5.17	4.84	5.96	0.34	4.64	0.09	3.78	5.18	4.66

B; IFNγ – CFA

	Th40			Conv. CD4		
Isotype	8.02	5.98	7.27	4.990	1.00	3.09
CD3	3.25	0.82	2.05	1.140	0.39	0.89
CD40	26.10	8.71	10.60	5.700	4.28	6.45
CD3+40	2.69	3.79	5.73	2.320	1.65	3.36
CD3+28	44.00	0.89	2.84	1.220	0.37	0.67
CD3+40+28	2.86	2.15	2.19	0.770	0.55	1.89

B; IFNγ – CFA+MOG

	Th40			Conv. CD4		
Isotype	3.570	5.23	2.99	0.800	1.38	0.60
CD3	1.470	3.03	1.27	0.680	0.84	0.28
CD40	16.200	8.56	14.60	7.640	5.20	5.34
CD3+40	0.027	3.89	2.64	0.034	2.26	0.96
CD3+28	5.630	1.60	1.46	4.660	0.98	1.20
CD3+40+28	1.430	1.19	2.12	0.840	0.66	1.38

B; IL-2 – CFA

	Th40			Conv. CD4		
Isotype	3.38	1.40	2.84	2.580	0.34	1.270
CD3	0.47	0.80	1.09	0.120	0.29	0.068
CD40	7.29	5.67	4.38	1.550	3.20	2.790
CD3+40	0.82	1.17	1.44	0.150	0.65	0.840
CD3+28	0.55	0.17	0.50	0.380	0.12	0.220
CD3+40+28	0.48	0.77	0.80	0.082	0.25	0.790

B; IL-2 – CFA+MOG

	Th40			Conv. CD4		
Isotype	0.37	2.59	1.39	0.14	0.67	0.28
CD3	0.40	1.17	0.54	0.14	0.51	0.15
CD40	3.34	2.23	3.57	1.16	1.82	1.33
CD3+40	0.90	2.46	1.58	0.82	1.84	0.69
CD3+28	0.21	0.30	0.38	0.00	0.14	0.19
CD3+40+28	0.30	0.40	0.49	0.33	0.00	0.17

B; TNFα – CFA

	Th40; CFA			Conv. CD4; CFA		
Isotype	1.72	1.37	1.84	1.99	0.40	0.88
CD3	8.27	6.55	9.06	17.80	16.40	18.00
CD40	2.23	0.60	1.13	1.90	0.57	1.75
CD3+40	16.00	8.03	13.10	27.30	13.10	18.90
CD3+28	11.80	4.20	7.98	29.90	16.00	28.60
CD3+40+28	14.50	10.50	10.10	33.60	32.70	30.60

B; TNFα – CFA+MOG

	Th40; CFA+MOG			Conv. CD4; CFA+MOG		
Isotype	1.06	1.26	0.70	0.52	0.36	0.49
CD3	10.50	12.20	5.57	26.70	30.40	14.50
CD40	0.87	0.75	0.92	1.12	1.39	0.68
CD3+40	4.82	4.43	6.62	11.30	9.52	10.10
CD3+28	14.30	6.38	8.05	34.50	16.00	20.70
CD3+40+28	7.83	7.61	10.20	21.80	14.80	26.90

Figure 5. (continued)

B; IL-17 – CFA

	Th40			Conv. CD4		
Isotype	1.07	0.50	0.71	1.16	0.58	0.58
CD3	5.67	6.96	8.39	11.70	21.90	20.90
CD40	0.49	0.38	0.55	1.05	0.94	0.82
CD3+40	10.90	6.20	8.17	22.30	14.10	15.60
CD3+28	7.69	3.57	6.27	24.00	17.90	26.10
CD3+40+28	6.11	5.62	9.85	20.10	19.90	32.20

B; IL-17 – CFA+MOG

	Th40			Conv. CD4		
Isotype	0.50	0.74	0.44	0.41	0.53	0.46
CD3	9.64	8.46	4.09	26.30	24.70	11.20
CD40	0.21	0.27	0.58	0.44	0.75	0.43
CD3+40	3.82	4.15	6.36	9.37	13.00	11.20
CD3+28	4.23	5.62	4.49	15.90	13.90	11.90
CD3+40+28	5.63	5.66	6.65	18.60	14.70	20.30

B; IL-10 – CFA

	Th40			Conv. CD4		
Isotype	0.31	0.46	0.37	0.51	0.31	0.22
CD3	4.88	6.40	7.41	9.94	18.20	15.80
CD40	0.17	0.43	0.11	0.81	0.54	0.34
CD3+40	8.61	3.60	5.17	20.40	9.35	12.10
CD3+28	6.27	5.27	4.84	16.60	19.80	17.00
CD3+40+28	6.29	3.98	5.96	20.90	14.80	18.80

B; IL-10 – CFA+MOG

	Th40			Conv. CD4	
Isotype	0.20	0.64	0.34	0.75	0.32
CD3	6.02	8.88	4.64	18.10	10.50
CD40	0.57	0.12	0.09	1.14	0.23
CD3+40	2.23	3.34	3.78	4.32	6.11
CD3+28	5.91	4.71	5.18	20.80	15.00
CD3+40+28	4.81	3.94	4.66	15.20	12.90

Figure 6.

A

	CFA			EAE			Un-induced
Va2	9.0	7.20	8.0	15.0	12.5	17.0	3.0
Va3.2	12.2	8.00	7.0	17.0	21.0	19.0	4.5
Va8	7.6	4.70	3.5	6.1	4.1	5.4	3.8
V8.3	10.9	10.21	10.3	10.2	11.0	10.0	5.0
Va11	11.9	9.20	4.7	10.0	8.0	9.0	2.0

B

	CFA			EAE			Un-induced
Vb2	7.9	5.4	9.0	9.0	10.0	7.9	5.90
Vb3	10.8	8.0	5.0	15.0	15.3	14.0	3.80
Vb4	7.5	8.0	9.0	16.0	18.0	17.0	6.00
Vb5	9.0	9.3	7.0	20.0	23.0	22.0	6.70
Vb6	12.0	8.0	5.0	12.0	8.0	6.2	6.40
Vb7	12.0	8.0	9.0	17.8	21.0	18.0	7.00
Vb8	4.7	2.2	3.2	3.5	4.0	2.0	8.20
Vb9	14.0	9.0	10.0	21.6	23.0	20.0	7.10
Vb10	16.0	19.0	17.0	21.0	17.0	18.0	6.80
Vb11	15.0	9.5	11.0	12.0	13.0	9.0	5.67
Vb13	6.7	5.1	5.8	7.0	6.7	9.0	5.70
Vb14	16.0	16.1	15.0	16.0	14.0	16.9	3.40
Vb17	18.0	14.0	12.0	17.0	14.0	11.0	4.30

C

	CFA			MOG	
Va2	5.2	10.0	5.0	8.8	5.1
Va3.2	12.0	7.1	8.0	9.2	5.4
Va8	8.0	2.9	3.0	2.5	0.5
Va8.3	10.7	4.3	4.5	2.6	1.2
Va11	12.5	8.6	3.7	5.3	11.3

D

	CFA			MOG		
Vb2	4.40	1.40	2.0	2.90	6.00	2.0
Vb3	13.40	5.00	4.3	5.80	3.50	5.0
Vb4	8.70	5.00	7.6	11.40	7.70	8.7
Vb5	7.80	4.90	4.2	16.00	5.90	9.0
Vb6	6.60	4.10	3.8	4.10	6.20	5.5
Vb7	8.60	5.60	9.2	8.30	11.10	12.0
Vb8	9.80					
Vb9	7.00	4.00	1.9	5.00	12.50	9.5
Vb11	7.80	4.50	5.1	20.00	19.00	12.0
Vb12	5.90	3.30	5.2	18.00	15.00	9.0
Vb13	16.40	4.20	3.3	10.90	5.20	8.5
Vb14	6.70	7.30	9.0	8.60	32.70	8.9
Vb17	16.50	15.70	11.0	8.60	21.70	20.0

Figure 7.

A; disease score

Day	Th40 cells + CFA			Th40 cells +CFA/MOG/PT			Th40 cells + PT			Th40 cells alone			Th40 cells + IFA/MOG/PT		
1	0.	0.	0.	0.	0.	0.	0.	0.	0.	0.	0.	0.	0.	0.	0.
2	0.	0.	0.	0.	0.	0.	0.	0.	0.	0.	0.	0.	0.	0.	0.
3	0.	0.	0.	0.	0.	0.	0.	0.	0.	0.	0.	0.	0.	0.	0.
4	0.	0.	0.	0.	0.	0.	0.	0.	0.	0.	0.	0.	0.	0.	0.
5	0.	0.	0.	0.	0.	0.	0.	0.	0.	0.	0.	0.	0.	0.	0.
6	0.	0.	0.	0.	0.	0.	0.	0.	0.	0.	0.	0.	0.	0.	0.
7	0.	0.	0.	0.	0.	0.	0.	0.	0.	0.	0.	0.	0.	0.	0.
8	0.	0.	0.	0.	0.	0.	0.	0.	0.	0.	0.	0.	0.	0.	0.
9	0.	0.	0.	0.	0.	0.	0.	0.	0.	0.	0.	0.	0.	0.	0.
10	0.	0.	0.	0.	0.	0.	0.	0.	0.	0.	0.	0.	0.	0.	0.
11	0.	0.	0.	0.	0.	0.	0.	0.	0.	0.	0.	0.	0.	0.	0.
12	0.	0.	0.	0.	0.	0.	0.	0.	0.	0.	0.	0.	0.	0.	0.
13	0.	0.	0.	0.	0.	0.	0.	0.	0.	0.	0.	0.	0.	0.	0.
14	0.	0.	0.	0.	0.	0.	0.	0.	0.	0.	0.	0.	0.	0.	0.
15	3.	0.	0.	0.	0.	0.	0.	0.	0.	0.	0.	0.	0.	0.	0.
16	3.	2.	3.	0.	0.	0.	0.	0.	0.	0.	0.	0.	0.	0.	0.
17	3.	2.	1.	0.	0.	0.	0.	0.	0.	0.	0.	0.	0.	0.	0.
18	3.	2.	1.	0.	0.	0.	0.	0.	0.	0.	0.	0.	0.	0.	0.
19	4.	2.	2.	0.	0.	0.	0.	0.	0.	0.	0.	0.	0.	0.	0.
20	4.	3.	4.	1.	0.	0.	0.	0.	0.	0.	0.	0.	0.	0.	0.
21	4.	4.	4.	2.	0.	0.	0.	0.	0.	0.	0.	0.	0.	0.	0.
22	4.	4.	4.	3.	0.	0.	0.	0.	0.	0.	0.	0.	0.	0.	0.
23	4.	4.	4.	4.	0.	0.	0.	0.	0.	0.	0.	0.	0.	0.	0.
24	4.	4.	4.	4.	1.	0.	0.	0.	0.	0.	0.	0.	0.	0.	0.
25	4.	4.	4.	4.	2.	0.	0.	0.	0.	0.	0.	0.	0.	0.	0.
26	4.	4.	4.	4.	2.	0.	0.	0.	0.	0.	0.	0.	0.	0.	0.
27	4.	4.	4.	4.	2.	0.	0.	0.	0.	0.	0.	0.	0.	0.	0.
28	4.	4.	4.	4.	3.	0.	0.	0.	0.	0.	0.	0.	0.	0.	0.
29	4.	4.	4.	4.	3.	0.	0.	0.	0.	0.	0.	0.	0.	0.	0.
30	4.	4.	4.	4.	4.	1.	0.	0.	0.	0.	0.	0.	1.	0.	0.
31	4.	4.	4.	4.	4.	0.	0.	0.	0.	0.	0.	0.	4.	2.	0.
32	4.	4.	4.	4.	4.	0.	0.	0.	0.	0.	0.	0.	5.	2.	0.
33	4.	4.	4.	4.	4.	0.	0.	0.	0.	0.	0.	0.	5.	2.	0.
34	4.	4.	4.	4.	4.	0.	0.	0.	0.	0.	0.	0.	5.	2.	0.
35	4.	4.	4.	4.	4.	0.	0.	0.	0.	0.	0.	0.	5.	3.	0.
36	4.	4.	4.	4.	4.	0.	0.	0.	0.	0.	0.	0.	5.	3.	0.
37	4.	4.	4.	4.	4.	0.	0.	0.	0.	0.	0.	0.	5.	3.	0.
38	4.	4.	4.	4.	4.	0.	0.	0.	0.	0.	0.	0.	5.	3.	0.
39	4.	4.	4.	4.	4.	0.	0.	0.	0.	0.	0.	0.	5.	3.	0.
40	4.	4.	4.	4.	4.	0.	0.	0.	0.	0.	0.	0.	5.	3.	0.
41	4.	4.	4.	4.	4.	0.	0.	0.	0.	0.	0.	0.	5.	3.	0.
42	4.	4.	4.	4.	4.	0.	0.	0.	0.	0.	0.	0.	5.	3.	0.
43	4.	4.	4.	4.	4.	0.	0.	0.	0.	0.	0.	0.	5.	3.	0.
44	4.	4.	4.	4.	4.	0.	0.	0.	0.	0.	0.	0.	5.	3.	0.
45	4.	4.	4.	4.	4.	0.	0.	0.	0.	0.	0.	0.	5.	3.	0.
46	4.	4.	4.	4.	4.	0.	0.	0.	0.	0.	0.	0.	5.	3.	0.
47	4.	4.	4.	4.	4.	0.	0.	0.	0.	0.	0.	0.	5.	3.	0.
48	4.	4.	4.	4.	4.	0.	0.	0.	0.	0.	0.	0.	5.	3.	0.
49	4.	4.	4.	4.	4.	0.	0.	0.	0.	0.	0.	0.	5.	3.	0.

B; disease score

Day	Th40+CFA						Th40 only				CD4hi-eff+CFA						CD4hi-eff only			
1	0.	0.	0.	0.	0.	0.	0.	0.	0.	0.	0.	0.	0.	0.	0.	0.	0.	0.	0.	0.
2	0.	0.	0.	0.	0.	0.	0.	0.	0.	0.	0.	0.	0.	0.	0.	0.	0.	0.	0.	0.
3	0.	0.	0.	0.	0.	0.	0.	0.	0.	0.	0.	0.	0.	0.	0.	0.	0.	0.	0.	0.
4	0.	0.	0.	0.	0.	0.	0.	0.	0.	0.	0.	0.	0.	0.	0.	0.	0.	0.	0.	0.
5	0.	0.	0.	0.	0.	0.	0.	0.	0.	0.	0.	0.	0.	0.	0.	0.	0.	0.	0.	0.
6	0.	0.	0.	0.	0.	0.	0.	0.	0.	0.	0.	0.	0.	0.	0.	0.	0.	0.	0.	0.
7	0.	0.	0.	0.	0.	0.	0.	0.	0.	0.	0.	0.	0.	0.	0.	0.	0.	0.	0.	0.
8	0.	0.	0.	0.	0.	0.	0.	0.	0.	0.	0.	0.	0.	0.	0.	0.	0.	0.	0.	0.
9	0.	0.	0.	0.	0.	0.	0.	0.	0.	0.	0.	0.	0.	0.	0.	0.	0.	0.	0.	0.
10	0.	0.	0.	0.	0.	0.	0.	0.	0.	0.	0.	0.	0.	0.	0.	0.	0.	0.	0.	0.
11	0.	0.	0.	0.	0.	0.	0.	0.	0.	0.	0.	0.	0.	0.	0.	0.	0.	0.	0.	0.
12	0.	0.	0.	0.	0.	0.	0.	0.	0.	0.	0.	0.	0.	0.	0.	0.	0.	0.	0.	0.
13	0.	0.	0.	0.	0.	0.	0.	0.	0.	0.	0.	0.	0.	0.	0.	0.	0.	0.	0.	0.
14	0.	0.	0.	0.	0.	0.	0.	0.	0.	0.	0.	0.	0.	0.	0.	0.	0.	0.	0.	0.
15	0.	0.	0.	0.	0.	0.	0.	0.	0.	0.	0.	0.	0.	0.	0.	0.	0.	0.	0.	0.
16	0.	0.	0.	0.	0.	0.	0.	0.	0.	0.	1.	0.	0.	0.	0.	0.	0.	0.	0.	0.
17	0.	0.	0.	0.	0.	0.	0.	0.	0.	0.	1.	0.	0.	0.	0.	0.	0.	0.	0.	0.
18	0.	0.	0.	0.	0.	0.	0.	0.	0.	0.	1.	0.	0.	0.	0.	0.	0.	0.	0.	0.
19	0.	0.	0.	0.	0.	0.	0.	0.	0.	0.	1.	0.	0.	0.	0.	0.	0.	0.	0.	0.
20	0.	0.	0.	0.	0.	0.	0.	0.	0.	0.	1.	0.	0.	0.	0.	0.	0.	0.	0.	0.
21	0.	0.	0.	0.	0.	0.	0.	0.	0.	0.	1.	0.	0.	0.	0.	0.	0.	0.	0.	0.
22	0.	0.	0.	0.	0.	0.	0.	0.	0.	0.	1.	0.	0.	0.	0.	0.	0.	0.	0.	0.
23	0.	0.	0.	0.	0.	0.	0.	0.	0.	0.	1.	0.	0.	0.	0.	0.	1.	0.	0.	0.
24	0.	0.	0.	0.	0.	0.	0.	0.	0.	0.	1.	0.	0.	0.	0.	0.	1.	0.	0.	0.
25	0.	0.	0.	0.	0.	0.	0.	0.	0.	0.	1.	0.	0.	0.	0.	0.	1.	0.	0.	0.
26	0.	0.	0.	0.	0.	0.	0.	0.	0.	0.	1.	0.	0.	0.	0.	0.	1.	0.	0.	0.
27	0.	0.	0.	0.	0.	0.	0.	0.	0.	0.	1.	0.	1.	0.	0.	0.	1.	0.	0.	0.
28	1.	0.	0.	0.	0.	0.	0.	0.	0.	0.	1.	0.	2.	0.	0.	0.	1.	0.	0.	0.
29	1.	0.	0.	0.	0.	0.	0.	0.	0.	0.	1.	0.	2.	0.	0.	1.	1.	0.	0.	0.
30	2.	0.	0.	0.	0.	0.	0.	0.	0.	0.	1.	0.	2.	0.	0.	1.	2.	0.	0.	0.
31	2.	0.	0.	0.	0.	0.	0.	0.	0.	0.	1.	0.	2.	0.	0.	1.	2.	0.	0.	0.
32	2.	0.	0.	0.	0.	0.	0.	0.	0.	0.	1.	0.	2.	0.	0.	1.	1.	0.	0.	0.
33	2.	0.	0.	0.	0.	0.	0.	0.	0.	0.	1.	0.	2.	0.	0.	1.	1.	0.	0.	0.
34	2.	0.	0.	0.	0.	0.	0.	0.	0.	0.	1.	0.	2.	0.	0.	1.	1.	0.	0.	0.
35	2.	0.	0.	0.	0.	0.	0.	0.	0.	0.	1.	0.	1.	0.	0.	1.	1.	0.	0.	0.
36	2.	0.	0.	0.	0.	0.	0.	0.	0.	0.	1.	0.	1.	0.	0.	1.	1.	0.	0.	0.
37	2.	1.	0.	0.	0.	0.	0.	0.	0.	0.	1.	0.	1.	0.	0.	1.	1.	0.	0.	0.
38	2.	1.	0.	1.	1.	1.	0.	0.	0.	1.	2.	1.	2.	1.	0.	1.	1.	0.	0.	0.
39	2.	1.	0.	1.	1.	1.	0.	0.	0.	1.	2.	1.	2.	1.	0.	1.	1.	0.	0.	0.
40	2.	4.	0.	1.	1.	1.	0.	0.	0.	1.	2.	1.	2.	1.	0.	1.	1.	0.	0.	0.
41	2.	4.	0.	3.	1.	1.	1.	0.	0.	1.	2.	1.	1.	2.	0.	1.	1.	0.	0.	0.
42	2.	4.	0.	4.	1.	1.	2.	0.	0.	1.	2.	1.	2.	2.	0.	1.	1.	0.	0.	0.
43	2.	4.	0.	4.	1.	2.	2.	0.	0.	1.	2.	1.	1.	1.	0.	1.	1.	0.	0.	0.
44	2.	4.	0.	4.	1.	2.	2.	0.	0.	0.	2.	1.	1.	1.	0.	1.	1.	0.	0.	0.
45	2.	4.	0.	4.	1.	1.	1.	0.	0.	0.	2.	0.	1.	2.	0.	2.	1.	0.	0.	0.
46	2.	4.	0.	4.	2.	1.	1.	0.	0.	0.	2.	0.	1.	2.	0.	2.	1.	0.	0.	0.
47	2.	4.	0.	4.	2.	1.	1.	0.	0.	0.	2.	0.	1.	2.	0.	2.	1.	0.	0.	0.
48	2.	4.	0.	4.	2.	1.	1.	0.	0.	0.	2.	0.	1.	2.	0.	2.	1.	0.	0.	0.
49	2.	4.	0.	4.	2.	0.	1.	0.	1.	0.	2.	0.	1.	1.	0.	2.	1.	0.	0.	0.
50	2.	4.	0.	4.	2.	1.	1.	0.	1.	0.	2.	0.	1.	2.	1.	2.	1.	0.	0.	0.
51	2.	4.	0.	4.	2.	1.	1.	0.	1.	0.	2.	1.	1.	2.	1.	2.	1.	0.	0.	0.
52	2.	4.	0.	4.	2.	1.	2.	0.	1.	0.	2.	1.	1.	1.	0.	1.	0.	0.	0.	0.
53	2.	4.	0.	4.	2.	1.	2.	0.	1.	0.	2.	1.	1.	0.	0.	1.	0.	0.	0.	0.
54	2.	4.	0.	4.	2.	1.	2.	0.	1.	0.	2.	1.	1.	0.	0.	1.	0.	0.	0.	0.
55	2.	4.	0.	4.	2.	1.	2.	0.	1.	0.	2.	1.	1.	0.	0.	1.	0.	0.	0.	0.
56	2.	4.	0.	4.	2.	1.	2.	0.	1.	0.	2.	1.	1.	0.	0.	2.	0.	0.	1.	0.
57	2.	4.	0.	4.	2.	1.	2.	0.	1.	0.	2.	1.	1.	0.	0.	2.	1.	0.	0.	0.
58	2.	4.	0.	4.	2.	2.	1.	0.	1.	0.	2.	1.	1.	0.	0.	1.	0.	0.	0.	0.
59	2.	4.	0.	4.	1.	1.	1.	0.	1.	0.	2.	1.	1.	1.	0.	1.	0.	0.	0.	0.
60	2.	4.	0.	4.	1.	1.	1.	0.	1.	0.	2.	1.	1.	1.	0.	1.	0.	0.	0.	0.
61	2.	4.	0.	4.	1.	1.	1.	0.	1.	0.	2.	1.	1.	1.	0.	1.	0.	0.	0.	0.
62	2.	4.	0.	4.	1.	1.	1.	0.	1.	0.	2.	1.	1.	1.	0.	1.	0.	0.	0.	0.
63	2.	4.	0.	4.	2.	1.	1.	0.	1.	0.	2.	1.	1.	1.	0.	1.	0.	0.	0.	0.
64	2.	4.	0.	4.	2.	2.	1.	0.	1.	0.	2.	1.	1.	1.	0.	1.	0.	0.	0.	0.
65	2.	4.	1.	4.	2.	2.	1.	0.	1.	0.	2.	1.	1.	2.	0.	1.	1.	0.	0.	0.
66	2.	4.	1.	4.	2.	2.	1.	0.	0.	0.	2.	1.	1.	2.	0.	1.	1.	0.	0.	0.

Figure 7. (continued)

D

	CD4+CD40+ Transfer										CD4+CD40- Transfer		
dLN	28.30	26.60	17.40	32.00	7.72	24.3	19.70	20.40	17.20	20.80	4.68	13.60	23.1
Spleen	2.79	2.66	1.64	1.54	4.36	3.3	3.46	3.22	3.09	2.49	8.84	9.46	18.7

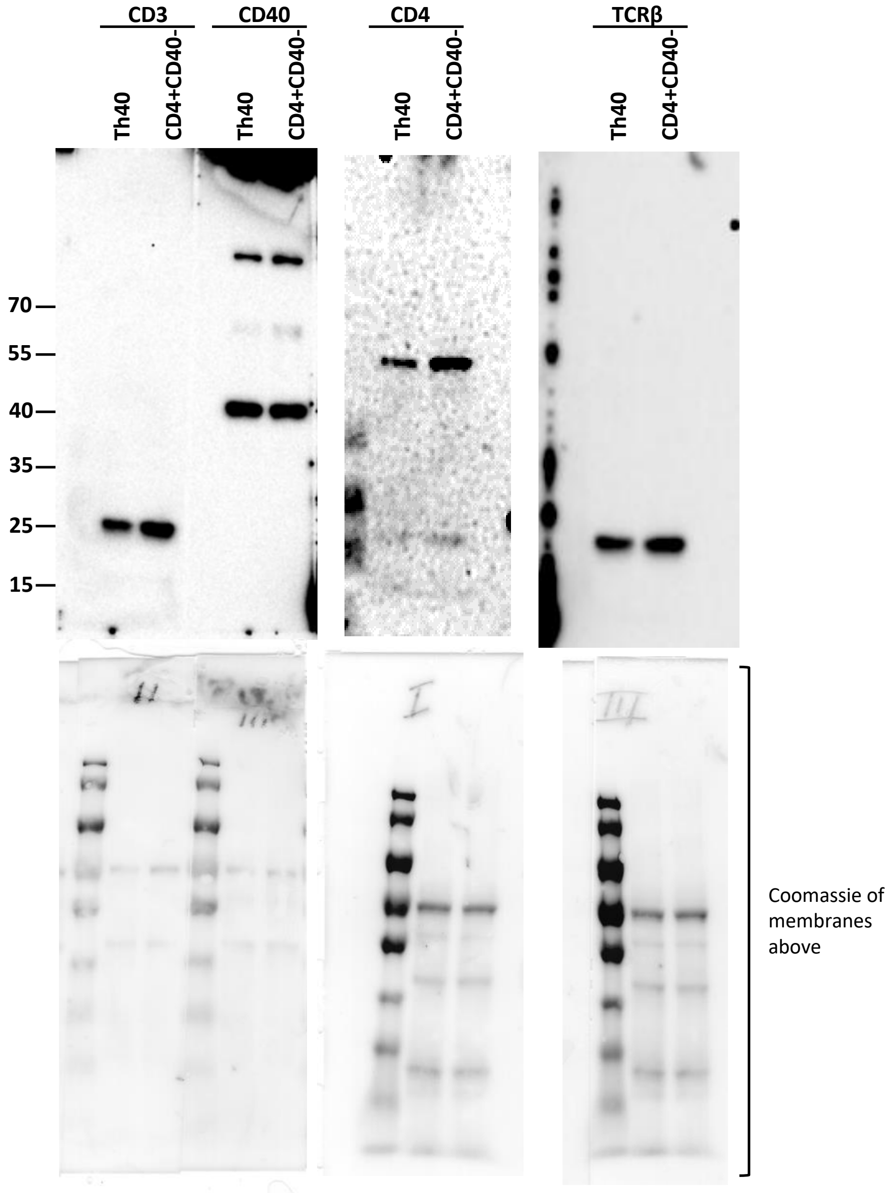

Figure 8.

Images in figure 8 are shown un-cropped in the actual figure.

S1 Fig.

Day	EAE in B6 Taconic				
1	0.	0.	0.	0.	0.
2	0.	0.	0.	0.	0.
3	0.	0.	0.	0.	0.
4	0.	0.	0.	0.	0.
5	0.	0.	0.	0.	0.
6	0.	0.	0.	0.	0.
7	0.	0.	0.	0.	0.
8	0.	0.	0.	0.	0.
9	0.	0.	0.	0.	0.
10	0.	0.	0.	0.	0.
11	1.	0.	0.	0.	0.
12	2.	0.	0.	0.	0.
13	4.	0.	0.	0.	0.
14	4.	0.	2.	1.	2.
15	4.	0.	2.	1.	2.
16	4.	2.	2.	2.	3.
17	4.	3.	2.	2.	3.
18	4.	3.	2.	2.	3.
19	4.	3.	3.	2.	3.
20	4.	3.	3.	2.	3.
21	4.	3.	3.	2.	3.
22	4.	3.	3.	2.	3.

S2 Fig.

Con. CD3	Con. CD4	Con. TCRb	EAE CD3	EAE CD4	EAE TCRb
85.7	65.9	84.6	62.6	93.1	91.5
83.6	64.1	81.5	50.6	93.6	91.9
77.1	67.5	82.3	55.7	92.9	89.5

S3 Fig.

CD4 percentage; Brain

Control	CFA 3d	CFA 7d	CFA 12d	MOG 3d	MOG 7d	MOG 12d	LPS	poly I:C
66.1	36.3	41.5	46.7	31.2	45.7	40.8	38.5	46.5
62.5	34.3	46.3	40.0	26.0	38.9	42.1	35.9	42.4
58.5	30.8	46.2	37.6	38.0	35.5	41.4	31.9	45.7

CD4 percentage; Spinal Cord

Control	CFA 3d	CFA 7d	CFA 12d	MOG 3d	MOG 7d	MOG 12d	LPS	poly I:C
47.60	32.60	29.40	31.50	24.80	26.40	29.70	29.7	32.10
47.40	30.40	35.30	54.40	29.00	41.10	32.60	23.2	27.80
48.30	34.70	45.90	35.30	34.30		42.80	30.1	26.40

CD4 cell numbers; Brain

Control	CFA 3d	CFA 7d	CFA 12d	MOG 3d	MOG 7d	MOG 12d	LPS	poly I:C
3.60	1.50	2.90	8.00	1.10	2.20	7.20	0.86	1.50
4.10	1.10	3.20	6.20	0.93	1.60	7.40	0.65	3.20
4.10	0.94	3.20	5.80	1.30	1.60	6.90	1.10	2.50

CD4 cell numbers; Spinal Cord

Control	CFA 3d	CFA 7d	CFA 12d	MOG 3d	MOG 7d	MOG 12d	LPS	poly I:C
0.24	0.57	0.51	0.74	0.97	0.62	1.10	1.20	2.80
0.64	0.54	0.73	1.40	1.20	1.10	1.10	3.00	1.20
0.58	0.62	0.89	0.80	1.30		1.50	3.00	0.83

CD8 percentage; Brain

Control	CFA 3d	CFA 7d	CFA 12d	MOG 3d	MOG 7d	MOG 12d	LPS	poly I:C
35.80	26.50	32.30	39.20	23.90	30.80	24.40	39.00	43.30
23.30	29.80	34.40	27.90	19.40	23.00	25.20	34.50	31.80
23.30	25.50	29.50	23.40	31.80	14.70	25.20	28.90	31.50

CD8 percentage; Spinal Cord

Control	CFA 3d	CFA 7d	CFA 12d	MOG 3d	MOG 7d	MOG 12d	LPS	poly I:C
47.20	29.10	42.20	46.40	18.80	39.50	40.40	29.90	47.50
44.20	26.70	47.90	61.20	24.10	55.30	39.70	18.90	44.00
46.60	30.80	55.70	45.90	21.40		51.10	23.80	47.20

CD8 cell numbers; Brain

Control	CFA 3d	CFA 7d	CFA 12d	MOG 3d	MOG 7d	MOG 12d	LPS	poly I:C
1.90	1.10	2.20	6.70	0.82	1.50	4.30	0.87	1.40
1.50	1.00	2.40	4.30	0.69	0.98	4.40	0.63	2.40
1.60	0.77	2.00	3.60	1.10	0.65	4.20	1.00	1.70

CD8 cell numbers; Spinal Cord

Control	CFA 3d	CFA 7d	CFA 12d	MOG 3d	MOG 7d	MOG 12d	LPS	poly I:C
0.24	0.51	0.73	1.10	0.74	0.92	1.70	1.20	4.20
0.59	0.47	0.99	1.60	0.97	1.50	1.40	2.50	1.90
0.56	0.55	1.10	1.00	0.83		1.80	2.40	1.50

CD40 in CD8; Brain

Control	CFA 3d	CFA 7d	CFA 12d	MOG 3d	MOG 7d	MOG 12d	LPS	poly I:C
24.20	32.40	44.50	48.10	8.60	35.90	57.60	48.20	44.70
22.50	22.00	40.50	63.70	23.90	32.50	54.60	49.70	53.50
27.60	19.20	30.20	64.30	15.90	33.60	53.70	45.50	49.80

CD40 in CD8; Spinal Cord

Control	CFA 3d	CFA 7d	CFA 12d	MOG 3d	MOG 7d	MOG 12d	LPS	poly I:C
24.20	65.40	47.10	56.30	44.90	36.50	61.10	55.70	55.50
22.50	61.60	61.40	36.70	46.40	48.50	62.90	67.40	57.30
27.60	68.10	40.70	46.20	48.90		49.20	59.20	88.90

S4 Fig.

A

Control	CFA 3d	CFA 7d	CFA 12d	MOG 3d	MOG 7d	MOG 12d	LPS	poly I:C
1.34	1.20	19.00	21.70	1.30	22.80	21.60	0.85	1.06
1.43	1.20	20.10	21.00	1.60	15.60	19.90	2.86	2.41
1.59	1.50	28.40	21.60	0.90	27.60	12.30	1.57	2.77
8.18								
6.86								

B

Control	CFA 3d	CFA 7d	CFA 12d	MOG 3d	MOG 7d	MOG 12d	LPS	poly I:C
3.34	2.80	49.50	47.10	2.50	40.10	52.40	1.100	3.00
4.82	2.70	36.60	50.00	3.10	35.30	55.40	5.740	1.09
4.36	4.00	55.60	49.90	10.90	47.90	60.20	8.540	2.54
8.17								
13.90								

C

Control	CFA 3d	CFA 7d	CFA 12d	MOG 3d	MOG 7d	MOG 12d	LPS	poly I:C
79.70	7.90	16.50	20.20	9.80	15.20	27.00	85.400	78.600
76.90	6.20	17.90	17.30	11.70	11.40	32.80	88.400	78.200
72.70	7.30	16.10	20.50	15.30	28.30	25.30	87.000	77.400
62.20								
69.10								

D

Control	CFA 3d	CFA 7d	CFA 12d	MOG 3d	MOG 7d	MOG 12d	LPS	poly I:C
88.80	14.40	66.30	58.70	25.70	51.10	71.80	84.600	86.700
86.50	12.10	51.40	65.10	30.50	45.40	59.60	94.600	67.400
86.80	19.20	64.50	64.40	31.50	60.90	63.70	94.800	60.900
77.90								
87.10								

S5 Fig.

Spleen; Total CD4

	Control					EAE			
Central Memory	32.5	30.1	31.4	33.5	26.7	35.6	31.7	38.7	44.0
Effector Memory	23.2	26.7	33.4	29.5	21.6	35.0	34.7	32.2	32.7

dLN; Total CD4

	Control					EAE			
Central Memory	16.50	23.80	15.60	27.00	15.30	31.2	38.2	36.0	29.9
Effector Memory	6.53	8.34	8.38	7.43	7.27	10.4	12.0	10.9	10.8

Spleen; Th40

	Control					EAE			
Central Memory	41.3	36.5	37.8	49.5	37.8	37.7	32.4	46.6	51.5
Effector Memory	29.2	32.4	36.9	34.9	35.5	44.4	45.2	42.0	39.2

dLN; Th40

	Control					EAE			
Central Memory	29.70	35.8	18.3	52.30	20.70	40.9	47.9	42.2	31.9
Effector Memory	9.44	14.1	14.0	9.86	19.70	15.6	16.8	12.9	16.5

S6 Fig.

	Th40+CFA recipients						Conv. CD4 recipients				
Brain	1.00	1.16	1.92	1.58	1.1		2.64	2.32	0.48	0.84	4.5
Spianl cord	1.88	1.64	0.52	1.68	0.7		1.24	1.64	1.04	0.52	4.1
