# Supplementary figures and images for: Th40 cells (CD4+CD40+ Tcells) drive a more severe form of Experimental Autoimmune Encephalomyelitis than conventional CD4 T cells
Source: PLoS One. 2017 Feb 13;12(2):e0172037. doi: 10.1371/journal.pone.0172037 (PMC5305068; doi:10.1371/journal.pone.0172037)

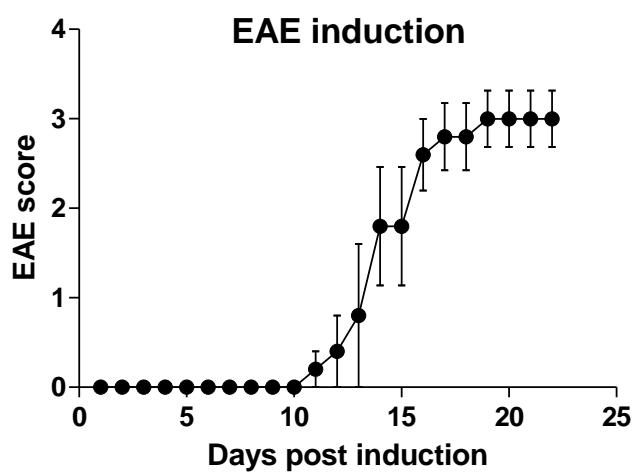

Supplement: S1 Fig — Mice (n = 5) were monitored for clinical disease scores. Average daily scores are shown. (PDF) [file pone.0172037.s002.pdf]

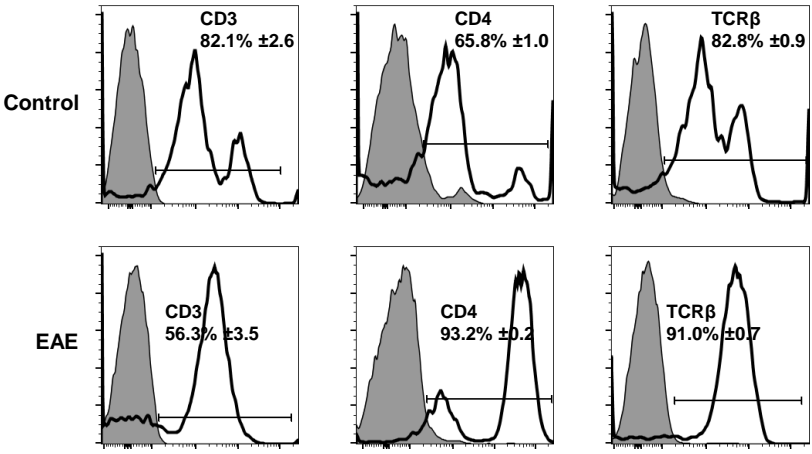

Supplement: S2 Fig — Tissue was homogenized to single cell suspension then mononuclear cells were purified on a 37%/70% discontinuous Percoll gradient. Resulting cells were washed then MHCII- and CD11b-expressing cells were depleted to remove B cells, macrophages and contaminating microglia cells. Cells were stained for surface expressed CD3, CD4, and TCRβ in flow cytometry. Gates were set based on isotype controls. Percent CD3 is from total live cells and CD4 and TCRβ are from total CD3 expressing cells. (PDF) [file pone.0172037.s003.pdf]

**A****Spleen CD69 in tot. CD4**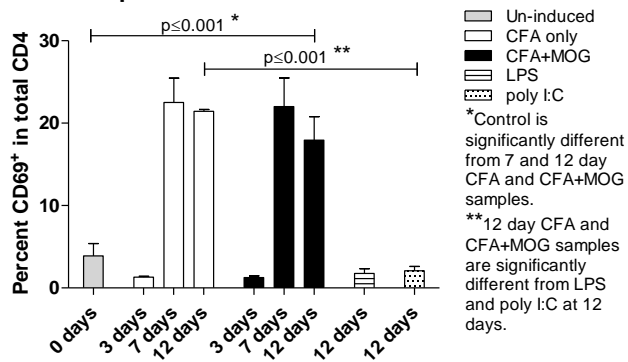**B****dLN CD69 in tot. CD4**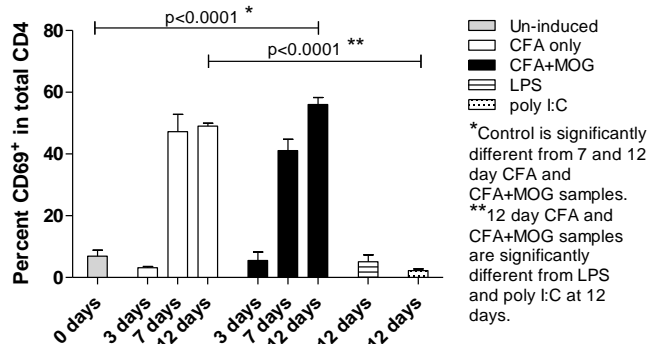**C****Spleen CD62L in tot. CD4**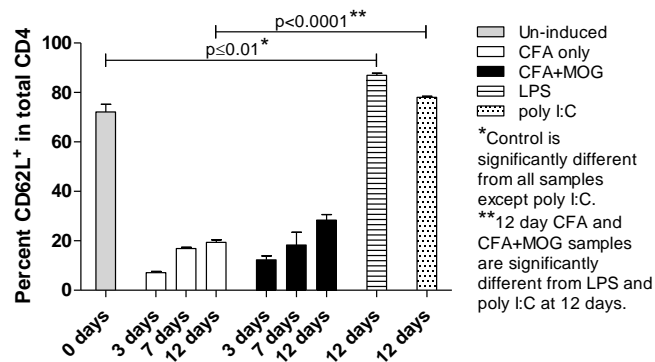**D****dLN CD62L in tot. CD4**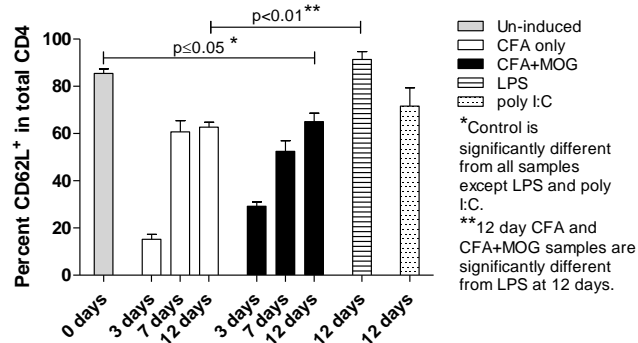

Supplement: S4 Fig — Alternatively, mice were challenged with LPS or poly I:C for 12 days or were left completely untreated (Un-induced). After 3, 7, and 12 days (n = 3 per time point in each group; disease scores for all mice was 0, except in the 3 CFA+MOG mice at 12 days where the scores were 2, 3, and 4 respectively) lymphocytes were purified from spleens and dLN and stained for CD4, CD40, CD69, and CD62L. All gates were set from isotype and FMO controls. CD69 and CD62L expression in total CD4 T cells was assessed in spleen (A and C respectively) and dLN (B and D respectively). Statistical differences were calculated by One-Way ANOVA with Bonferroni post-test. Data in figure are representative of 3 experiments. (PDF) [file pone.0172037.s005.pdf]

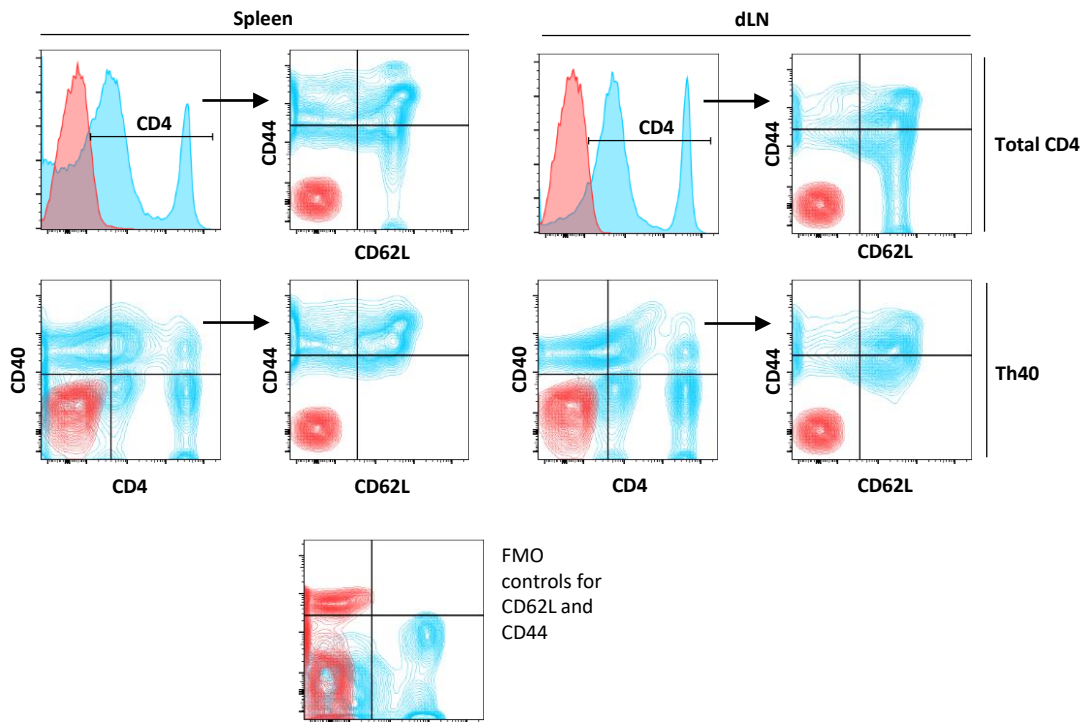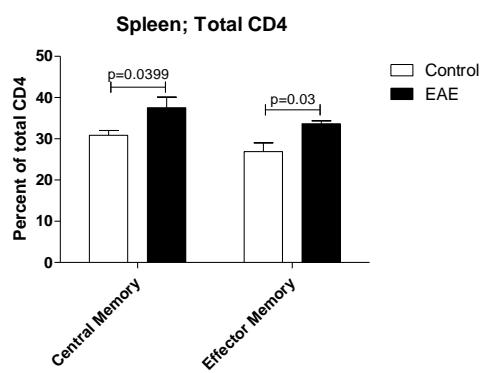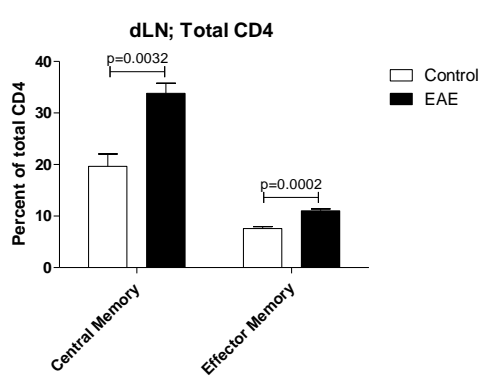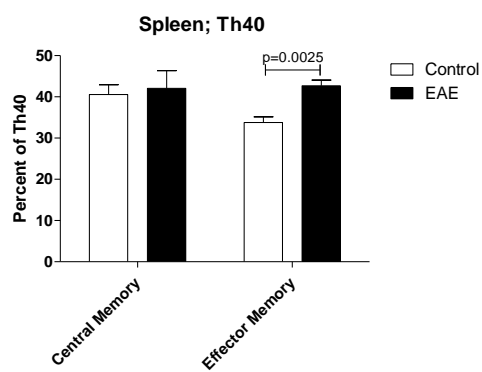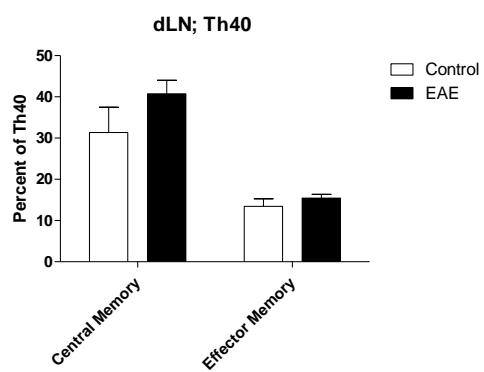

Supplement: S5 Fig — When the EAE induced mice reached a disease score of 2, 2, 3, and 3, respectively, spleens and dLN were harvested and stained for CD3, CD4, CD40, CD62L and CD44. Cells were gated on CD3 then central (CD62L+CD44+) and effector (CD62L-CD44+) memory cells were assessed within the total CD4 population and within the Th40 population. Gates were set based on isotype and FMO controls. Statistical difference was calculated by two-tailed t-test and significant differences are noted in the graphs. (PDF) [file pone.0172037.s006.pdf]

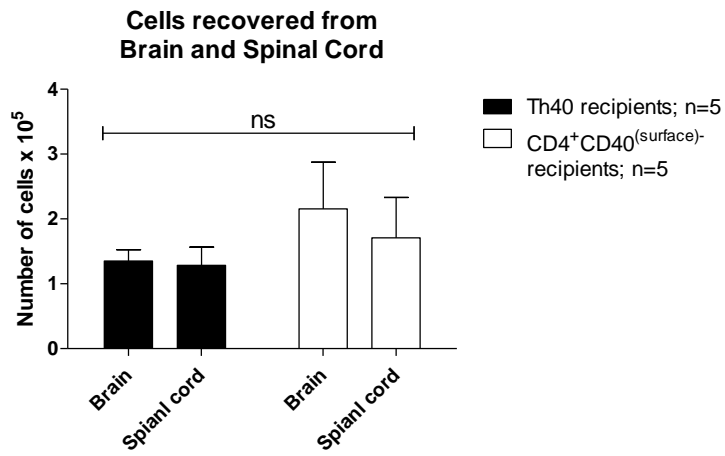

Supplement: S6 Fig — When Th40 recipients reached a disease score of at least 2, brains and spinal cords were harvested from those mice, as well as from the CD4+CD40(surface)- T cell recipients that were at lower scores at the same time points. T cells were purified and enumerated. Statistical difference was calculated by two-tailed t-test. (PDF) [file pone.0172037.s007.pdf]
