# Supplementary material for: Th40 cells (CD4+CD40+ Tcells) drive a more severe form of Experimental Autoimmune Encephalomyelitis than conventional CD4 T cells
Source: PLoS One. 2017 Feb 13;12(2):e0172037. doi: 10.1371/journal.pone.0172037 (PMC5305068; doi:10.1371/journal.pone.0172037)
Supplement: S3 Fig — Alternatively, mice were challenged with LPS or poly I:C. After 3, 7, and 12 days (n = 3 per time point in each group; disease scores for all mice was 0, except in the 3 CFA+MOG mice at 12 days where the scores were 2, 3, and 4 respectively) lymphocytes were purified from brains (left panels) and spinal cords (right panels) and stained for CD3, CD4, and CD40 for flow cytometry. Cells were first gated on CD3 then CD4 and CD40 levels were assessed in that gate. All gates were set from isotype controls. Percentages and numbers of total CD4 or CD8 T cells were calculated. Levels of CD40-expressing CD8 cells were determined. Statistical differences were calculated by One-Way ANOVA with Bonferroni post-test. Data in figure represent at least 3 experiments. (PDF) [file pone.0172037.s004.pdf]

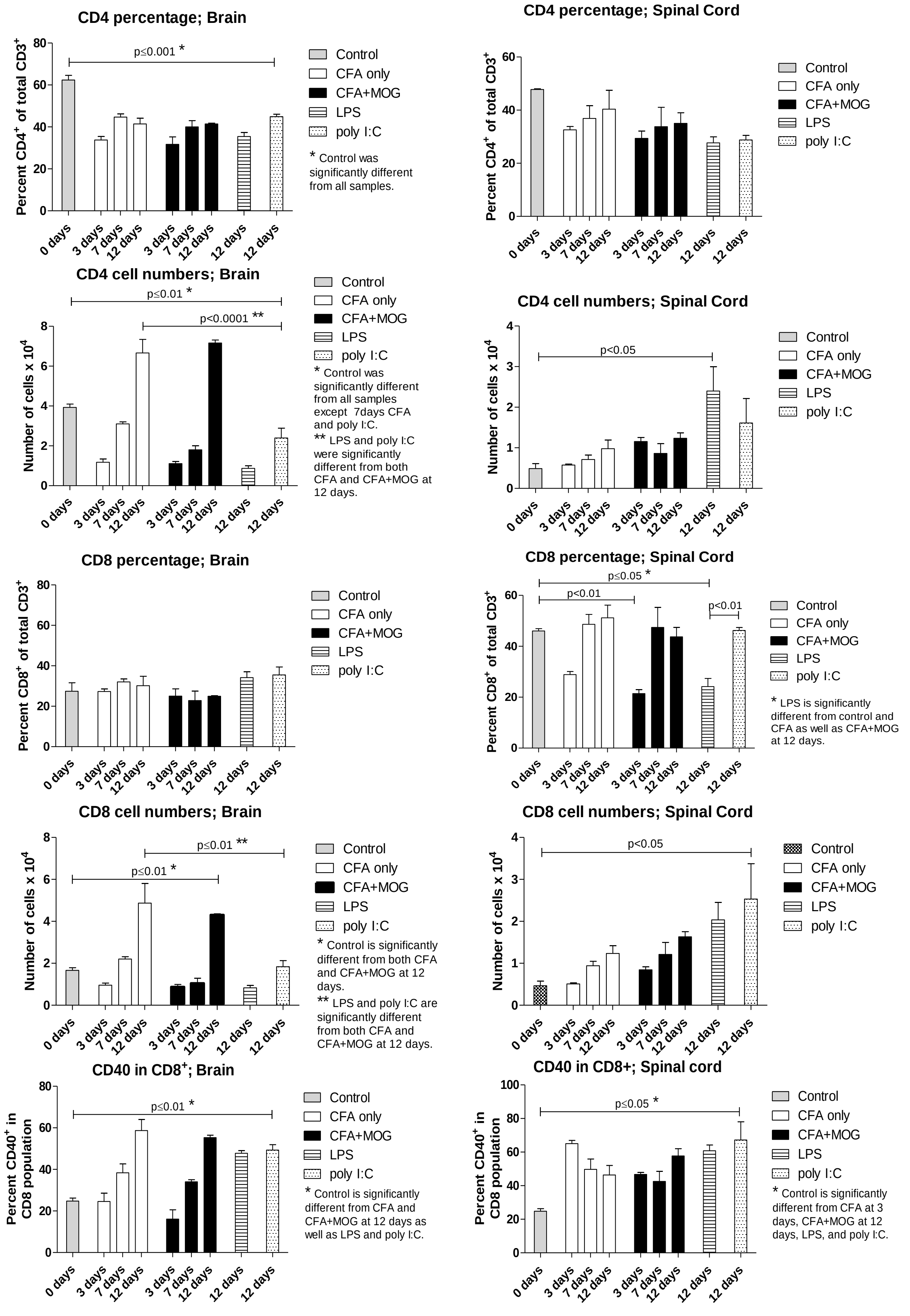
CD4 percentage; Brain
Percent CD4+ of total CD3+
p≤0.001 *
* Control was significantly different from all samples.
CD4 percentage; Spinal Cord
Percent CD4+ of total CD3+
CD4 cell numbers; Brain
Number of cells x 10^4
p≤0.01 *
p<0.0001 **
* Control was significantly different from all samples except 7days CFA and poly I:C.
** LPS and poly I:C were significantly different from both CFA and CFA+MOG at 12 days.
CD4 cell numbers; Spinal Cord
Number of cells x 10^4
p<0.05
CD8 percentage; Brain
Percent CD8+ of total CD3+
CD8 percentage; Spinal Cord
Percent CD8+ of total CD3+
p≤0.05 *
p<0.01
p<0.01
* LPS is significantly different from control and CFA as well as CFA+MOG at 12 days.
CD8 cell numbers; Brain
Number of cells x 10^4
p≤0.01 **
p≤0.01 *
* Control is significantly different from both CFA and CFA+MOG at 12 days.
** LPS and poly I:C are significantly different from both CFA and CFA+MOG at 12 days.
CD8 cell numbers; Spinal Cord
Number of cells x 10^4
p<0.05
CD40 in CD8+; Brain
Percent CD40+ in CD8 population
p≤0.01 *
* Control is significantly different from CFA and CFA+MOG at 12 days as well as LPS and poly I:C.
CD40 in CD8+; Spinal cord
Percent CD40+ in CD8 population
p≤0.05 *
* Control is significantly different from CFA at 3 days, CFA+MOG at 12 days, LPS, and poly I:C.
Control
CFA only
CFA+MOG
LPS
poly I:C
0 days
3 days
7 days
12 days
